# Supplemental vibrotactile feedback control of stabilization and reaching actions of the arm using limb state and position error encodings

**DOI:** 10.1186/s12984-017-0248-8

**Published:** 2017-05-02

**Authors:** Alexis R. Krueger, Psiche Giannoni, Valay Shah, Maura Casadio, Robert A. Scheidt

**Affiliations:** 1 Biomedical Engineering, Marquette University, Milwaukee, WI USA; 2 Informatics, Bioengineering, Robotics and Systems Engineering, University of Genova, Genoa, Italy; 3 Robotics, Brain and Cognitive Science, Italian Institute of Technology, Genoa, Italy; 4 Physical Medicine and Rehabilitation, Northwestern University Feinberg School of Medicine, Chicago, IL USA; 5 Sensory Motor Performance Program, Rehabilitation Institute of Chicago, Chicago, IL USA; 6 Neurology, Medical College of Wisconsin, Wauwatosa, WI USA; 7 Neuromotor Control Laboratory, Department of Biomedical Engineering, Marquette University, Olin Engineering Center, 206, P.O. Box 1881, Milwaukee, WI 53201-1881 USA

**Keywords:** Sensory substitution, Sensory augmentation, Stroke, Proprioception, Kinesthesia

## Abstract

**Background:**

Deficits of kinesthesia (limb position and movement sensation) commonly limit sensorimotor function and its recovery after neuromotor injury. Sensory substitution technologies providing synthetic kinesthetic feedback might re-establish or enhance closed-loop control of goal-directed behaviors in people with impaired kinesthesia.

**Methods:**

As a first step toward this goal, we evaluated the ability of unimpaired people to use vibrotactile sensory substitution to enhance stabilization and reaching tasks. Through two experiments, we compared the objective and subjective utility of two forms of supplemental feedback – limb state information or hand position error – to eliminate hand position drift, which develops naturally during stabilization tasks after removing visual feedback.

**Results:**

Experiment 1 optimized the encoding of limb state feedback; the best form included hand position and velocity information, but was weighted much more heavily toward position feedback. Upon comparing optimal limb state feedback vs. hand position error feedback in Experiment 2, we found both encoding schemes capable of enhancing stabilization and reach performance in the absence of vision. However, error encoding yielded superior outcomes - objective and subjective - due to the additional task-relevant information it contains.

**Conclusions:**

The results of this study have established the immediate utility and relative merits of two forms of vibrotactile kinesthetic feedback in enhancing stabilization and reaching actions performed with the arm and hand in neurotypical people. These findings can guide future development of vibrotactile sensory substitution technologies for improving sensorimotor function after neuromotor injury in survivors who retain motor capacity, but lack proprioceptive integrity in their more affected arm.

**Electronic supplementary material:**

The online version of this article (doi:10.1186/s12984-017-0248-8) contains supplementary material, which is available to authorized users.

## Background

Kinesthesia refers to sensations of limb position and movement [5] derived predominantly from information encoded by muscle spindle afferents, which are sensitive to muscle length and rate of length change (c.f., [39]). Deficits of kinesthetic feedback are common after stroke. Almost 50% of stroke survivors experience impaired limb position sense in their contralesional arm [9, 11, 15]. Loss of kinesthetic sensation contributes to impaired control of reaching and stabilization behaviors that are vital to an independent life style [6, 45, 56, 64]. Although people suffering loss of kinesthetic feedback can move by relying on vision of their limbs, long processing delays inherent to the visual system (100–200 ms; [8]) yield movements that are typically slow, poorly-coordinated, and require great concentration [18, 41]. Visually guided corrections come too late and result in jerky, unstable movements [42]. Unfortunately, many stroke survivors give up using their contralesional limb because of their sensorimotor deficits [55] even though this reduces quality of life [1, 56].

Our long-term goal is to mitigate the negative impact of post-stroke kinesthesia deficits by creating sensory substitution technologies that provide real-time feedback of contralesional arm state (e.g., the position and velocity of the arm and hand) to a site on the body retaining somatosensation (e.g., the ipsilesional arm). As a first step, the current study tested the ability of people with no known neuromotor deficits to control goal-directed actions using supplemental vibrotactile stimuli that provided real-time feedback about the moving limb to a part of the body that was not itself involved either in the movement or in essential behaviors like speaking and eating. We justify this approach by noting that the vast majority of people – including neurologically intact individuals - exhibit imperfect somatosensory control of the arm and hand in the absence of ongoing visual feedback. Indeed, the most conspicuous and ubiquitous manifestation of imperfect somatosensation is “proprioceptive drift” ([60]; c.f., [52]), wherein marked errors in the perceived position of the unseen hand develop within a period of 12 to 15 s [36]. Proprioceptive error is likely due to a progressive drift between visual and proprioceptive maps of body configuration when vision of the relative positions of the body and the visual target is precluded [23]. Proprioceptive drift succinctly predicts the pattern of performance errors observed during goal-directed reaching [43] and stabilizing actions [53] performed with the hand in the absence of visual feedback.

The idea of providing supplemental feedback to mitigate sensory deficits has been explored for many decades (c.f., [61]). Successful applications include cochlear implants (c.f., [31]) and non-invasive systems that encode video images into either vibratory or electrical signals applied to the skin at one of several body parts (abdomen, back, thigh, fingertip, forehead, tongue) [25]. Vibrotactile systems for enhancing postural stabilization in vestibular patients have been proposed [28, 50] and show promise [37] when the synthesized feedback includes all task-relevant states [27]. Vibrotactile systems also show promise for providing information about grasp force and hand aperture to users of myoelectric forearm prostheses [63]. While these past works reveal the brain’s remarkable ability to integrate synthetic feedback for perception and control, shortcomings include a lack of focus on limb kinesthesia and use of feedback systems that have potential to negatively impact quality of life, for example by interfering with verbal conversation (cf., [2]). Several research groups have designed supplemental feedback systems for enhancing training of arm movements in healthy individuals by stimulating the moving limb ([3, 4, 30]; for a review see [49]), but those systems could not be effective in individuals suffering somatosensory deficits in the more affected limb. We therefore propose to use vibrotactile stimulation of one arm to deliver supplemental kinesthetic feedback pertaining to motion of the other arm. We rationalize this choice because, aside from the palms and fingers, tactile feedback from the surface of the arm does not appear to be critically important for completing most daily living activities and thus, we minimize the likelihood that the vibrotactile display would impede use of the stimulated arm for other tasks.

There are many conceivable ways to encode information about a moving limb within a vibrotactile feedback stream, and it is unclear which way might best facilitate closed-loop control of goal-directed stabilizing and reaching behaviors. One possibility is the encoding of limb state (e.g., the position and/or velocity of the moving hand). From the perspective of technological implementation, the hardware and software tools needed to develop stand-alone wearable technologies capable to detect, synthesize and deliver limb state information in unconstrained environments are readily available. A second, distinct approach, “goal-aware” feedback [58], additionally encodes information about the current task’s objectives. For example, a simple form of goal aware vibrotactile feedback might indicate the instantaneous error between the hand’s current position and the position of a visual target. A more complex version might encode information about which direction to move the arm, based on the output of a computational model implementing an optimal trade-off between kinematic and energetic performance. Although goal-aware feedback might yield better performance than limb state feedback because it includes additional task-specific information, this approach suffers from a number of unique technological challenges that state feedback avoids. In particular, determining the user’s motor intentions and movement goals from one moment to the next in a dynamically changing environment seems to be a daunting undertaking. Errors in estimating intent would lead to unreliable feedback, thus compromising usability of the vibrotactile display for daily life applications.

Human physiology provides no clear guidance on how kinesthetic information might be encoded within supplemental vibrotactile feedback to optimize augmented closed-loop control of stabilization and reaching behaviors. For example, muscle spindle primary endings encode muscle length and the rate of length change in a joint-based coordinate reference frame whereas muscle spindle secondary endings encode primarily muscle length information [39]. Simulated vibrotactile limb state feedback could readily emulate these types of native feedback. By contrast, Golgi tendon organs encode muscle tension [39], which may be more difficult to estimate and emulate. Additionally, γ-motor neurons can modulate the sensitivity of muscle spindles in ways that are - in some cases - suggestive of error encoding [21], although the functional dependence of spindle feedback on γ-motor neuron activity is rather complex [19].

**Fig. 1:**
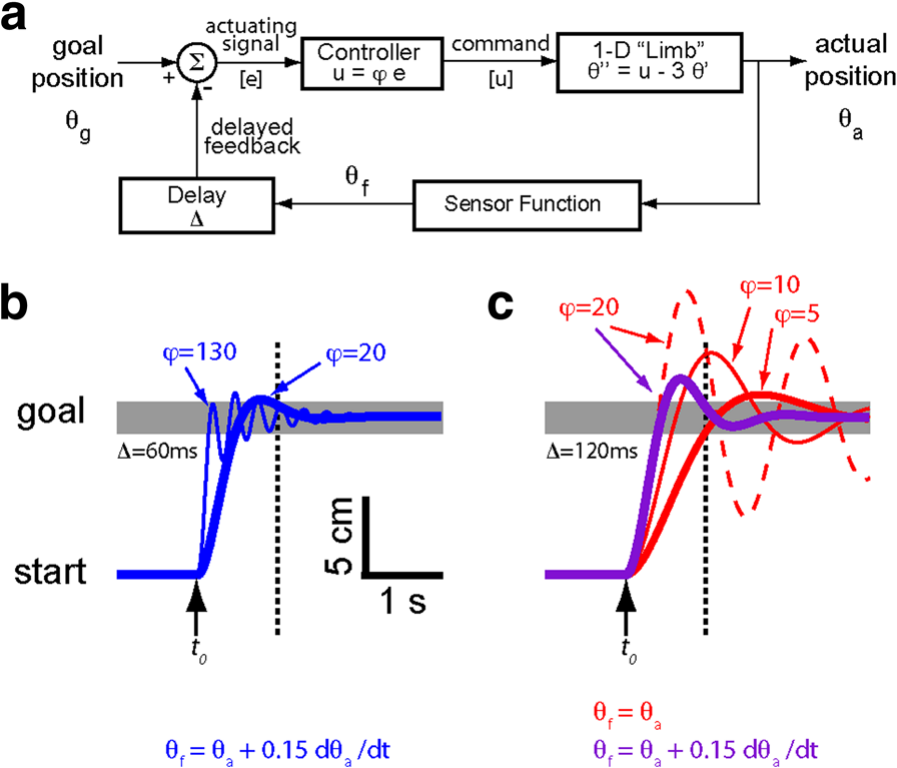
Simplified model of closed-loop feedback control for goal-directed reaching. **a** Simplified model demonstrating how feedback delay (∆) and information content (Sensor Function) impacts performance of a proportional controller regulating the position θ_a_ of a damped inertial “limb” modeled as a second order differential equation relating changes in limb kinematics (position, velocity and acceleration) to changes in the control input u. Controller gain φ was varied to test the capabilities of the model system. **b** Simulation results of limb displacement (*vertical axis*) plotted as a function of time (*horizontal axis*) when the feedback path emulates proprioception (i.e., Delay ∆ = 0.06 s and Sensor Function θ_f_ = θ_a_ + 0.15 dθ_a_/dt). *Arrow* indicates the time of change in desired position (depicted in arbitrary units of displacement). Dotted line: t = 1 s. *Grey band*: goal target zone. The limb obtains the goal within the time constraint over a broad range of controller gains with position + velocity feedback (*Thick blue trace*: φ = 20; *Thin trace*: φ = 130). **c** Simulated reaching under visual guidance (*Red*: Delay ∆ = 0.12 s and Sensor Function θ_f_ = θ_a_; *Thick red trace*: φ = 5; *Thin trace*: φ = 10; *dashed trace*: φ = 20). With position feedback, no value of φ enables success when ∆ = 0.12 s. Also shown (*Purple*; φ = 20) is an acceptable solution obtained when simulated visual feedback also includes velocity information: θ_a_ + 0.15 dθ_a_/dt. For panels **b** and **c**, the horizontal scale bar depicts 1 s whereas the vertical scale bar represents 5 cm displacement

To gain some insight on possible kinesthetic encoding schemes, consider a simplified, single-joint model of a human-in-the-loop state feedback control system (Fig. 1a). Although this model is not intended to replicate the complexities of human sensorimotor control (for example see [20]), it includes many characteristics relevant to the current problem: a “limb” dominated by viscoinertial dynamics; feedback delay arising from sensory transduction, transmission and processing; and a central mechanism that transforms performance errors into corrective motor commands, modeled here using a simple proportional control law. We justify use of proportional control because we ultimately seek to design a sensory substitution system that requires minimal information processing by the stroke-injured brain.

Consider now a task wherein the limb should acquire and hold a goal target in less than 1 s (Fig. 1b, vertical dotted line). If feedback emulates the dynamics and delay associated with muscle spindle afferents [i.e., position plus velocity feedback and a sensory delay of ~60 ms; [8]], a wide range of controller gain values (φ, ranging from 20 to 130) can yield acceptable performance (Fig. 1b, blue traces). Thus, position plus velocity feedback can yield robust performance that is relatively insensitive to controller gain while requiring minimal computational load (i.e., implementing a simple proportional control law). In the absence of reliable proprioceptive feedback (e.g., post-stroke), one might be inclined to substitute visual feedback. The model suggests that relying solely on hand position feedback relative to a fixated target with a visual delay of ~120 ms [8] cannot yield acceptable capture-and-hold performance in this task for any proportional gain value (Fig. 1c, red traces). Even if the limb reaches the target within 1 s, the hold criteria is subsequently violated. Acceptable performance can be restored only by making the feedback and/or controller more complex (e.g., with position plus velocity feedback encoding; Fig. 1c, purple trace).

This study sought to determine how best to synthesize and deliver supplemental kinesthetic feedback and to test its ability to enhance performance of goal-directed stabilization and reaching behaviors in neurotypical adults. In a first set of experiments, we evaluated different weighted combination of moving hand position and velocity information to find the form of supplemental state feedback that minimizes performance error during stabilization and reaching tasks performed with the arm. Based on the predictions of the computer model, we hypothesized that a vibrotactile feedback encoding scheme that includes a modest amount of hand velocity information - but weighted more heavily toward position information - would best impact performance of these behaviors. In a second set of experiments, we compared this optimal limb state feedback to hand position error feedback [one of the simplest forms of “goal aware” feedback [58]] to determine the performance benefits of each encoding scheme. We hypothesized that both state and error feedback would be capable of enhancing performance of stabilization and reaching behaviors in the absence of visual feedback. We furthermore hypothesized that error encoding would likely yield superior enhancement of these behaviors due to the additional task-relevant information that error feedback contains. Preliminary aspects of this study have been presented in abstract form [26].

## Methods

Twenty-six healthy humans (13 female) were recruited from the University of Genoa community and all provided written informed consent to participate in this study. All procedures were approved by local Institutional Review Boards serving the University of Genoa (ASL3 Genovese) and Marquette University in accord with the 1964 Declaration of Helsinki. None of the participants had known neurological disorders. Participant ages ranged from 22 to 32 (26 ± 3) years. All of the participants self-reported to be right handed. All participants had normal or corrected-to-normal vision and all were naïve to the purposes of the study.

Each participant took part in up to three experimental sessions conducted on separate days. The experiments were designed to determine whether encoding state or error information about a moving limb (e.g., the dominant arm) into a vibrotactile feedback stream applied to a non-moving body part (e.g., the non-dominant arm) would best enhance performance of stabilization and reaching behaviors in the absence of ongoing visual feedback of performance. Specifically, the first session (Experiment 1) sought to determine the best (optimal) combination of limb state information, hand position and velocity feedback, to encode within the vibrotactile feedback applied to the contralateral arm. The purpose of the second and third sessions (Experiment 2) was to compare the effects of encoding optimal state feedback with that of encoding an objective measure of hand position error.

### Experimental setup

**Fig. 2:**
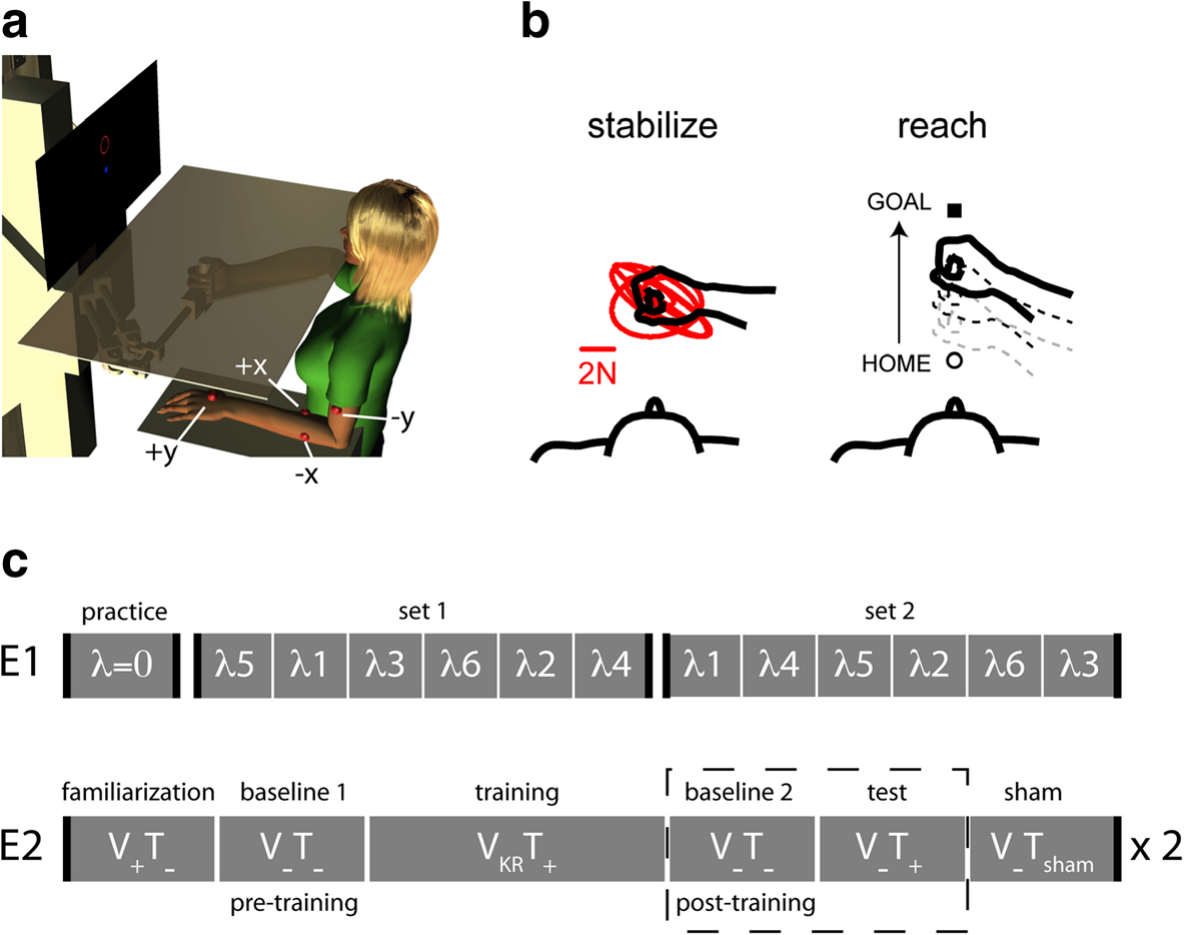
Experimental setup and protocol. **a** Participant at robot holding the end effector of a planar manipulandum, with integrated forearm support. An opaque screen occluded direct visual feedback of task performance; the left arm shows the standard placement of the four tactors (*red dots*). **b** Tasks. *Left*: stabilizing the hand at a fixed point in space against robotic perturbations. *Right*: example of a center-out reaching movement. **c** Sequence of events in each experiment. E1: Experiment 1. E2: Experiment 2; baseline 2 and test 2 were counter balanced in order across participants. Visual feedback (V) and vibrotactile feedback (T) was either continuous (+), absent (−), or only used for providing the results at the end of each task (KR). This sequence was used during 2 sessions, in which the only difference was that the vibration feedback encoded either error or state

Participants were seated comfortably in a high-backed, adjustable-height chair in front of a horizontal planar robotic manipulandum, which has been described in detail previously (see [10]) (Fig. 2a). The participant was seated approximately 25 cm from the center of the workspace with the right arm strapped to the robotic handle and to its integrated forearm support. The arm support consisted of a lightweight rigid bar attached to the robot’s handle through a bearing that allowed free horizontal planar rotation of the hand + forearm about the center of rotation of the handle. The seat height was adjusted such that the abduction angle of the right shoulder was between 75° and 85°. The left arm rested comfortably on a horizontal planar armrest situated below that plane of motion of the robot; the forearm and hand pointed forward as in Fig. 2a. An opaque shield was placed over the workspace to block the participant’s view of the moving arm and the robotic apparatus. View of the stationary arm was not precluded. A vertical computer monitor was mounted in direct view, 0.7 m in front of the participant and just above the shield to avoid neck strain; this display provided visual cues of hand and target position and motion when appropriate (the scheduling of visual feedback is described below).

**Fig. 3:**
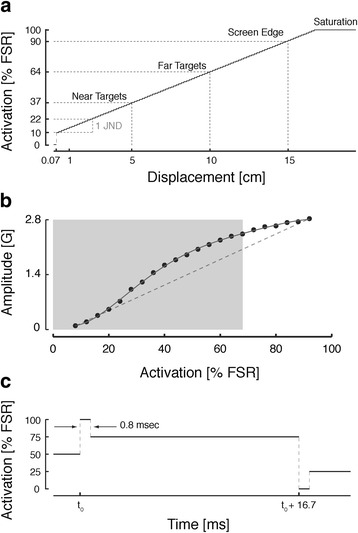
Tactor activation characteristics. **a** Exemplar activation mapping for state feedback of hand position wherein displacements of the hand were mapped onto tactor excitation voltages, as a percentage of Full Scale Range (FSR = 5.0 V). **b** Realized mapping between tactor activation and vibration amplitude (data points), and a *solid line* representing the best-fit polynomial reveal a nonlinear, monotonically increasing relationship over the entire half-workspace encoded by each tactor. A *dashed line* fit between the lowest and highest sample points provides an “ideal” linear reference for comparison. *Grey* shading indicates the half-workspace spanned by the home and far targets. **c** Pulse-step scheme employed to decrease the response time of the tactors. See text for details

Supplemental kinesthetic feedback was provided using a two-channel (4 “tactor”) vibrotactile display attached to the non-moving arm. Each tactor consisted of a micro-motor with integrated eccentric rotating mass (Pico Vibe 10 mm vibration motors; Precision Microdrives Inc., Model # 310–117), with an operational frequency range of 50 to 250 Hz and peak vibrational amplitude of 2.8G, which corresponded to an expected maximal forearm-plus-hand acceleration ranging between 0.53 m/s^2^ and 0.77 m/s^2^ depending on participant anthropometrics. We employed a nonlinear activation map (Fig. 3a). The intensity of vibration was 100% full scale when activation was 5.0 V, vibration was OFF when activation was 0.0 V, and above a minimum level of activation (0.5 V), the intensity of vibration increased monotonically with activation voltage (Fig. 3b). The frequency of vibration in Hz was roughly 100 times the amplitude of vibration in G. Below the minimum level of activation, nonlinear “stiction” within the motors prevented the motors from engaging. To improve tactor response time and the bandwidth of frequency modulation, we also employed a “pulse-step” activation strategy, whereby a desired tactor activation level was implemented by first activating the tactor fully (100% activation) for 0.8 milliseconds before reducing activation to the desired level (Fig. 3c). This control scheme also increased the repeatability of tactor excitation levels, especially at the lowest levels of activation, where nonlinear stiction was most noticeable.

For all participants, tactors were initially arranged with one tactor on the back of the hand, two tactors on the forearm, and one on the upper arm (Fig. 2a; default tactor locations indicated by red spheres). In this standard configuration, the hand tactor (+y tactor) was placed approximately 1 cm proximal to the first and second finger metacarpophalangeal joints. The forearm tactors were placed approximately 3 cm distal to the cubital fossa, one on each side of the forearm (+x tactor on the right, −x tactor on the left). The upper arm tactor (−y tactor) was placed on the bicep muscle belly about 5 cm proximal to the cubital fossa. Elastic fabric bands were used to secure the tactors. This default tactor configuration was designed such that inter-tactor spacing exceeded two-point discrimination thresholds for dermatomal regions of the arm and forearm, as reported by Nolan [33].

We then performed a verification procedure wherein we adjusted tactor locations slightly so that each participant could indicate reliably which tactor or pair of tactors was activated at any given time. This was done using low, middle, and high intensity vibrations (approximately 10%, 50% and 90% full scale range, respectively). The adjustment/verification procedure began by asking the participant to place the hand’s cursor at each of the four corners of the screen and to tell the experimenter how many and which tactors were vibrating (at ~90% FSR). This was repeated two times, once adjacent to the center of the screen (~10% FSR) and again approximately mid-way between the center and the edge of the screen (~50% FSR). Next, the participant was asked to place the hand’s cursor at the middle of the screen, and then to move away from and back towards the center so as to feel the changing intensity. If the participant could not give clear and correct indication as to which tactor was active and the appropriate direction of activation change, further personalized tests were used to isolate and resolve the problem. This setup procedure successfully identified well-discriminated stimulation sites in all participants and typically took between 5 and 10 min to complete. However, it should also be noted that for 16 of our healthy participants, finding well-discriminated sites did require adjustments (see Discussion).

The vibrotactile display was calibrated to the robot’s workspace such that motions of the robot handle to the right would induce the + X tactor to vibrate, whereas motions of the robot handle away from the participant (i.e., toward the monitor) would induce the + Y tactor to vibrate. The various mappings of hand kinematics onto vibratory stimuli are described in greater detail below.

### Experimental tasks

Across the three days of testing, participants were required to perform two different experimental tasks (Fig. 2b). This included: i) stabilizing the hand at a fixed point in space against robotic perturbations; and ii) reaching to 16 spatial targets that sampled 16 movement directions and two movement extents.

#### Stabilizing

When performing the stabilization task, participants attempted to hold the robot’s handle steady at a comfortable “home” position located in the center (origin) of the workspace. During each 1 min stabilization trial, the robot generated spatially complex sum-of-sinusoid force perturbations that contained a low frequency component (0.25 Hz) and several high frequency components (1.1 Hz, 1.2 Hz, 1.65 Hz and 1.75 Hz) (Eq. 1a and 1b):

1a $$ {F}_X = 0.75\cdot cos\ \left(2\pi \cdot 1.75\cdot t\right) + 0.75\cdot cos\ \left(2\pi \cdot 1.2\cdot t\right) + 6\cdot cos\ \left(2\pi \cdot 0.25\cdot t\right) $$

1b $$ {F}_Y = 0.75\cdot sin\ \left(2\pi \cdot 1.65\cdot t\right) + 0.75\cdot sin\ \left(2\pi \cdot 1.1\cdot t\right) + 6\cdot sin\ \left(2\pi \cdot 0.25\cdot t\right) $$

During pilot testing, some individuals adopted a strategy whereby they stabilized hand position by stiffening the arm and ignored the vibrotactile feedback altogether. We therefore gave study participants the following instructions: “Without stiffening your arm, keep your hand as steady as possible using the vibration feedback.” Depending on the specific experimental test conditions, participants could perform the stabilization task under three different visual feedback conditions. In the first condition (continuous visual feedback; V_+_), a 0.5 cm radius cursor was always visible on the computer screen and tracked the motion of the hand continuously. In the second feedback condition (no visual feedback; V_−_), participants attempted to stabilize the hand at the home position without ongoing cursor feedback (i.e., the cursor was never visible). In the third visual feedback condition (Knowledge of Results; V_KR_), participants only received cursor feedback of final hand position (not the entire hand path) after the trial was complete. Reminders to avoid stiffening the arm and to focus on the vibration were repeated periodically throughout the stabilization trials.

#### Reaching

During this task, participants performed out-and-back reaches to 16 targets. For each of the targets, participants reached to the target, paused for a few seconds, and executed a return-to-home movement for a total of 32 discrete goal directed reaches. Each target-capture movement started from the same comfortable “home” position as in the stabilization task. The 16 spatial targets were equally distributed along the perimeter of two concentric circles that were centered on the home position (center target). Eight “near” targets were distributed around an inner circle that had a radius of 5 cm, whereas eight “far” targets were distributed around an outer circle that had a radius of 10 cm. We attempted to equalize the Fitts Law difficulty of the different movements by scaling the size of targets such that the inner targets had 1 cm radii whereas the outer targets had 2 cm radii. The presentation order of the targets was pseudorandomized within each block. As in the stabilization task, reaching trials could be conducted with one of the three different forms of visual feedback.

In the V_+_ case, a 0.5 cm radius cursor tracked the motion of the hand continuously. At the start of a trial, an 800 Hz audio cue sounded for 0.2 s, and one of the 16 visual targets was presented on the video display. When the cursor reached the target, the 800 Hz audio cue sounded again and the participant could relax. After a 2 s pause, the current target disappeared, a final 800 Hz audio cue sounded, the home target appeared, and the participant reached back to the starting position in anticipation of the next trial.

In the V_−_ trials, the cursor was never visible as participants attempted to capture the visual targets. At the start of these trials, the 800 Hz audio cue sounded and one of the 16 visual targets appeared. Upon completing the reach, the participant announced that they thought they had arrived to the target and the experimenter registered that event by pressing a button. At this point, the 800 Hz audio cue indicated the trial was complete regardless of the spatial accuracy of the movement. Following a 2 s pause, the current target disappeared, a final 800 Hz audio cue sounded, the home target appeared, and the participant reached back to the starting position. Once again, the participant verbally indicated completion of the movement and the experimenter registered the event in anticipation of the next trial.

In V_KR_ trials, participants received visual feedback of the final cursor/hand position only after the reach was complete; they were to use that feedback to correct for any terminal target capture errors. In this condition, the participant reached to the target without visual feedback as in the V_−_ case. When the participant announced that they had arrived to the target, the experimenter pressed the button and the cursor appeared. If the cursor was correctly on-target, the 800 Hz audio cue sounded. If the cursor was incorrectly off-target, an annoying, 2000 Hz to 400 Hz descending-pitched audio tone sounded for 1.5 s, and the participant was required to correct the error with the aid of visual feedback. Upon arriving on the target the pleasing 800 Hz audio cue sounded. After a 2 s pause, this sequence of events was repeated for the return-to-home reach.

In all three conditions, time constraints were placed on each reach. If the participant had not reached the target or announced to the experimenter they had reached the target within 10 s, the experimenter terminated the movement and the experiment proceeded as normal. In all cases, participants were instructed to “Capture the target as quickly and accurately as possible.” As a reminder to capture the target quickly, reach targets turned from red to blue 1 s after they appeared.

### Vibrotactile feedback encoding schemes

Hand position data derived from the robot’s optical encoders were used to generate three distinct forms of vibrotactile cues. The first, *state feedback,* was a weighted combination of hand position and velocity information. Position feedback was calibrated such that the intensity of vibrotactile feedback was zero in all tactors when the hand was centered on the home target (i.e., the origin of the workspace) and increased proportionally within the vibrotactile display as a vector representation of the hand’s deviation from that position. The vibration was 0 only when the cursor was at the center of the home target’s location. Vibratory stimulation reached 90% full-scale range (FSR: 75 Hz to 200 Hz) when the hand reached the bounds of the visual display (i.e., at a displacement of 15 cm from home along the positive and negative x and y axes). The distance between target circles (i.e., 5 cm) corresponded to a difference in vibration magnitudes greater than 2 *just noticeable differences* (JNDs) as reported in a psychophysical study of a vibrotactile discrimination using a similar vibrotactile display configuration [47, 48]. The bijective linear mapping between hand position and stimulation within the X-Y vibrotactile display adhered to the intuitive registration between the robotic and vibrotactile reference frames. Velocity feedback was calibrated such that the intensity of vibrotactile feedback was zero in all tactors when the hand was at rest, regardless of where the hand was in the workspace. Vibratory stimulation increased proportionally within the vibrotactile display as a vector representation of the hand’s instantaneous velocity. Vibratory stimulation reached 90% FSR when hand speed reached 20 cm/s. Prior to use, participants were instructed that the state feedback vibration encoding scheme provided position and velocity feedback information relative to the home position.

The second form of vibrotactile cue, position *error feedback*, was defined as the instantaneous signed error between the hand and target locations. Error feedback was calibrated as for position feedback, except that the origin of the vibrotactile display was always centered on the current target rather than always on the home position. The vibration was set to 0 if the cursor was anywhere within the current specified target. The sign and magnitude of error in each feedback channel (X or Y) determined which tactor within that channel was activated (+ or -) and the extent to which it was activated (stronger vibrations indicated larger errors). Prior to use, participants were instructed that this vibration feedback scheme provided information about the position error between the cursor and the target. Error feedback encoding is a form of “repulsive” feedback in the sense that to optimize performance, participants were to reduce the magnitude of vibration within the vibrotactile display.

The third form of vibrotactile feedback, *sham feedback*, was created by applying a Fourier transform to a selected vibrotactile feedback signal recorded during pilot testing from a participant performing a dynamic stabilization task while using error feedback. The phase of the selected feedback signal was scrambled in the frequency domain and inverse Fourier transformed, yielding a signal that maintained the power content of the original vibration signal within each frequency bin, but did not encode any information about either the hand position or the current task. Stabilization about the target produced a signal containing both high- and low- frequency changes in the vibration. By randomizing the signal’s phase in the frequency domain, the resulting sham signal retained a spectral power signature similar to that of both the reaching and stabilization tasks in both the state and error feedback conditions, but did not convey any information meaningfully related to ongoing task performance. The resulting sham signal was 1 min long and was looped during trials lasting longer than 1 min. The sham signal was only used at the end of an experimental session. Participants who noticed the onset of sham stimulation and voiced their awareness were instructed to nevertheless attempt to use the vibration as best as they could.

**Fig. 4:**
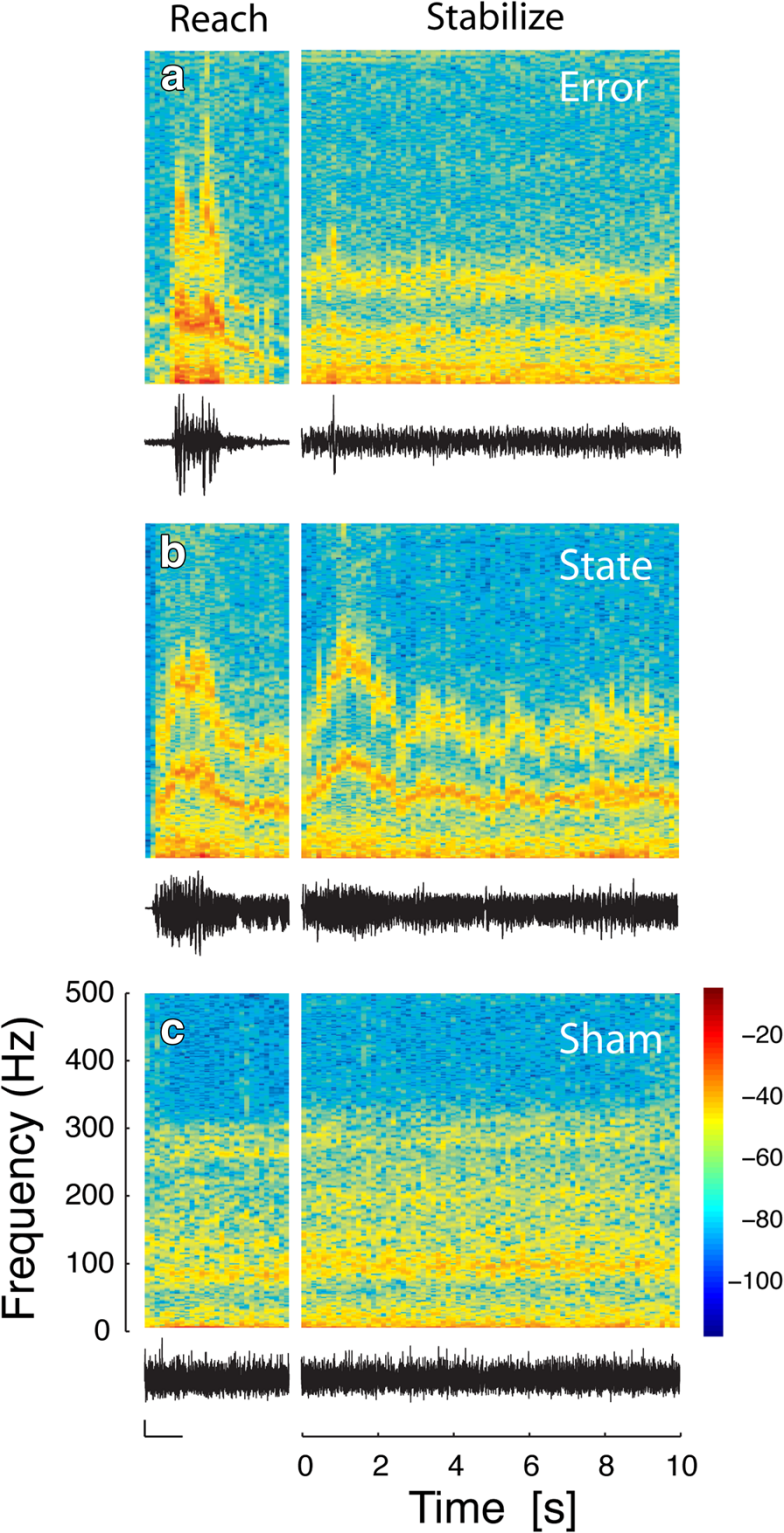
Vibrotactile information encoding schemes: spectrograms and time-aligned time series waveforms. **a** Error feedback encoding scheme. Time series (*bottom*) and spectrogram (*top*) of vibrotactile feedback during an exemplar reach (*left*) and a portion of a stabilization trial (*right*). Frequency axes and time scale as in panel (**c**). For the time series, the scale bars in the bottom panel (*left*) represent 0.1 G (*vertical axis*) and 1 s (*horizontal axis*). **b** Optimal state encoding scheme (λ = 0.2). **c** Sham feedback encoding scheme. See text for details. *Colorbar*: signal power relative to total signal power, in units of dB

Figure 4 presents representative waveforms and spectrograms derived from the vector magnitude time series of measured vibrotactile stimuli recorded within the vibrotactile display during selected reach and stabilization actions. With error feedback, vibrotactile stimuli tapers off as subjects acquire the reach target (Fig. 4a, left). During stabilization however, robotic perturbations induce systematic time-varying performance errors that give rise to relatively steady amounts of vibration in the vibrotactile display that are modulated by the ongoing performance (Fig. 4a, right). State feedback (Fig. 4b) gives rise to stronger end-of-reach vibrotactile stimulation in the 100 Hz to 200 Hz frequency band as subjects acquire the near and far targets, as well as throughout the stabilization trials. By contrast, sham feedback (Fig. 4c) stimulates the arm and hand with a spectral signature that closely replicates the average frequency content of the state and error feedback, albeit without temporal modulation meaningfully related to ongoing task performance.

### Experiment 1 - Optimizing state feedback

There currently exists no theoretical or empirical guidance on how best to combine information about a moving limb’s dynamic state when synthesizing and delivering supplemental kinesthetic feedback for promoting stable and accurate limb control in the absence of visual feedback. The first experiment used several different combinations of hand position and velocity information to systematically compare the utility of both forms of information to promote limb stability and movement accuracy in the absence of ongoing visual feedback.

Fifteen participants (9 female; age range 22 to 32 years) performed 12 matched pairs of one reaching trial (reaches to and from 16 different targets) and one stabilizing trial (1 min duration). Each pair of trials utilized one of six specific weighted combinations of hand position and velocity information (Eq. 2):

2 $$ \gamma (t)=\lambda \cdot \overset{.}{p}+\left(1-\lambda \right)\cdot p $$

where the vibrotatile feedback signal *γ*(*t*) is a vector function of hand position *p* and *ṗ* velocity. *λ* is a constant scalar weighting factor such that when *λ* = 0 feedback contained only position information, whereas when *λ* = 1, feedback contained only hand velocity information. The *λ* parameter varied from 0 to 1 in increments of 0.2. Each *λ* value was tested two times, one time in each of two blocks of 6 matched pairs. The presentation order of *λ* values was pseudorandomly distributed within each block (an example of one such randomization is depicted in Fig. 2c, E1).

At the start of the experimental session, participants were informed that the vibrotactile feedback could encode several different combinations of hand position and velocity information, ranging from pure position feedback to pure velocity feedback. They were then introduced to the vibrotactile display with position-only feedback (*λ*=0.0) and encouraged to freely explore the workspace to develop an understanding of how the vibration feedback interface worked. After free exploration, participants practiced the reaching task and then the stabilization task until they were comfortable with the vibrotactile display and the tasks. During the reaches, V_KR_ visual feedback was provided only after the movement was completed to allow for terminal correction of target capture errors. During the stabilizations, visual feedback was not available at all (i.e., the V_−_ condition). In both types of trials, participants were encouraged to use the vibration to complete the tasks to the best of their ability.

Participants then completed the 2 blocks of six matched trial pairs (one *λ* value per pair). Before assessing performance under each new encoding scheme, participants were again encouraged to freely explore the workspace to learn the relationship between vibration and hand position/velocity; they were allowed up to one minute to do so. Participants then completed the reaching trial followed by the stabilization trial. This exploration-reach-stabilize sequence was repeated for each *λ* value within each block. We designed this sequence of tasks such that the reach and return movements would provide structured practice with the current *λ* encoding scheme prior to performance testing in the stabilization task. At the end of each matched trial pair, participants were asked to describe, if possible, how they interpreted and used the vibration to perform each task. Although we originally intended the reaching task to serve as structured practice for stabilization, we report performance in both the stabilizing and reaching trials because we observed consistent results in both tasks.

### Experiment 2 - Comparison of optimal state vs. error feedback

We next compared effectiveness of two forms of supplemental vibrotactile feedback in guiding performance of stabilization and reaching behaviors in the absence of visual feedback. The first encoding scheme, optimal state feedback, was the best combination of limb position and velocity information identified in Experiment 1. The second scheme, error feedback, involved the encoding of performance error into the vibrotactile information stream. Error encoding is a simple form of “goal aware” feedback (c.f., [58]) wherein deviations from a desired position (the “goal”) are fed back to the user, who can drive performance back to that desired position. In this experiment, the desired position was defined simply as the instantaneous position of the target.

Fifteen participants (8 Female) participated in 2 experimental sessions at least 2 h apart (Range 2 h to 19 days; mean 5 ± 6 days). Both sessions followed the same experimental protocol but used a different type of feedback, *optimal limb state feedback* (as determined by the first experiment) or goal-aware *error feedback*. The presentation order of state and error feedback sessions was counterbalanced across participants. Four of these participants had also participated in Experiment 1.

During each session, participants completed a series of reaching and stabilization tasks guided by various combinations of visual (V) and vibrotactile (T) feedback (Fig. 2c, E2). First, participants familiarized on the tasks by performing each with continuous vision and without vibration feedback (V_+_T_−_). Participants repeated the tasks with neither visual nor vibration feedback (V_−_T_−_) to assess baseline performance before vibration training. Following baseline assessment, participants were introduced to the vibrotactile display and encouraged to freely explore the workspace for up to 3 min. Participants then received training throughout the workspace by performing five reaching trials with vibrotactile feedback and visual knowledge of results (V_KR_T_+_). Participants concluded training by performing the stabilization task with V_−_T_+_ feedback. On average, participants took 45 min to do this training. We then examined aftereffects of training by having participants complete both tasks without either visual or vibration feedback (V_−_T_−_) (i.e., post-training baseline testing). We also tested how well participants could use vibrotactile feedback to guide performance of reaching and stabilizing behaviors in the absence of vision by having them perform one trial of each task with only vibrotactile feedback (V_−_T_+_) (i.e., vibrotactile performance testing). The presentation order of the post-training baseline phase and the vibrotactile performance testing were counter balanced across participants (Fig. 2c, dashed box). Lastly, the participants performed both tasks with sham vibrotactile feedback (V_−_T_sham_). Subjects were provided brief intervals (1 to 2 min) of V_+_T_−_ feedback between each experimental phase, thus allowing periodic realignment of visual and proprioceptive maps of space.

At the end of each error or state feedback session, participants were asked to rate the “usefulness” of that particular encoding scheme on a scale that ranged from 1 to 7, by responding to three questions: “ How useful was the vibration in the {reaching, stabilization} task?”. They were asked to describe, if possible, how they interpreted and used the vibration to perform each task.

### Data analysis

Analysis of participant performance during stabilization and reaching behaviors focused primarily on hand position data, which were derived from the robot described in [10] using Python (Python Software Foundation) and H3D API (SenseGraphics, www.h3dapi.org) running at 60 Hz to collect data from the robot’s encoders and control the visual display.

Stabilizing: Conspicuous features of participant performance during stabilization trials included the presence of startup transients at the beginning of each trial and the presence of prolonged hand position “drift” in the absence of ongoing visual feedback (see also [43, 53, 60]). We therefore discarded the first 5 s of data in each 60-s trial to eliminate potential start-up transients caused by the onset of force perturbations applied to the hand. We modeled hand position drift along the x and y axes separately as low-order (linear through 3^rd^-order) polynomial functions of time. The purpose of this manipulation was to isolate variations in the data due to slow drift from moment-by-moment fluctuations caused by the robotic force perturbations of Eq. 1a and 1b. As the 2^nd^- and 3^rd^-order models yielded results indistinguishable from the linear model, we only report results obtained with the lowest order model. We then computed the root-mean-square error for the raw position data (RMSE_total_) as well as for the portions of data variance accounted for by the drift model (RMSE_drift_) and by the moment-by-moment fluctuations in the data (i.e., the residuals of the drift model fit, RMSE_residual_). For each component (RMSE_total_, RMSE_drift_, RMSE_residual_), we analyzed only the RMSE values in the second block of trials of six different lambda values performed by each participant. Then, we fit third order polynomials to the variations in RMSE_total_, RMSE_drift_, and RMSE_residual_ values across the population of participants. Finally, we identified the optimal mixture of limb state information within the vibrotactile feedback signal by identifying the lambda value that minimized the third order polynomial fit.

Reaching: Performance in the reaching task was quantified using hand positions sampled when the participant indicated that they thought they had acquired the intended targets. These final reach positions were used to compute two performance measures: *mean absolute error magnitude* (a measure of reach accuracy), and *target capture variability* (a measure of reach precision). Mean absolute error was computed separately for each intended target type {center, near, far} as the average root-mean-square error between the final reach position and the center of the intended target. Target capture variability was estimated separately for each intended target type by computing the area of the 95% confidence interval (CI) ellipse for the entire distribution of final hand positions about the desired target ([24, 35]; see also, [45]). For the near and far targets, we collapsed across movement directions by counter-rotating the reach endpoints about the home target by the angle of desired target movement. For the center target, we calculated target capture variability in two ways – after counter-rotating by the intended movement direction (as for the near and far targets), and without counter-rotation.

### Statistical analysis

This study tested three main hypotheses via two sets of experiments. The goal of Experiment 1 was to identify the form of vibrotactile limb state feedback (i.e., the specific weighted combination of moving hand position and velocity information) that elicits the best performance of stabilizing and reaching behaviors in the absence of ongoing visual feedback. Based on the predictions of a simple proportional control model, we hypothesized that a vibrotactile feedback encoding scheme that includes a modest amount of hand velocity information - but weighted more heavily toward position information - would best enhance performance of these behaviors. The goal of Experiment 2 was to perform a head-to-head comparison of optimal state feedback and hand position error feedback, which is a simple “goal-aware” encoding scheme (c.f., [58]). We hypothesized that both state and error feedback would enhance performance of stabilization and reaching behaviors in the absence of visual feedback. We furthermore hypothesized that error encoding would yield superior enhancement of these behaviors due to the additional task-relevant information contained in this encoding scheme.

Prior to statistical testing, each of the performance measures described above required correction for non-normality (i.e., skew) in their distributions, stemming from the fact that these measures are strictly non-negative. A Box-Cox transformation [${T}_{\lambda}(y)=\left({y}^{\lambda}-1\right)/\left(\lambda {\overline{y}}^{\lambda -1}\right)$] [7] was used to correct for distribution skew. Here, *y* is the variable to be transformed while $\lambda$ is a transformation parameter.

In Experiment 1, we used multivariate analysis of variance (MANOVA), followed by repeated measures ANOVA and Dunnett’s multiple comparison *t*-test (where appropriate) to determine the extent to which stabilization performances (RMSE_total_, RMSE_drift_, and RMSE_residual_) at each value varied relative to those at the optimal value, which was identified by minimizing the polynomial fit to the population RMSE_drift_ data. For reaching, we similarly analyzed mean absolute error and target capture variability for each target type {center, near, far}. We calculated the within-subject difference between performances at each value relative to the performances measured at the optimal value identified during stabilization. We then performed a 1-sample *t*-test to evaluate the statistical significance of the population differences (H_0_: difference = 0.0).

In Experiment 2, we tested the ability of vibrotactile feedback to enhance performance of stabilizing. First we quantified the amount of the mean absolute error magnitude accounted for by drift in each of the various training conditions. Then we analyzed the effect of the experimental phase {post-training baseline V_−_T_−_, test V_−_T_+_, sham V_−_T_sham_} and feedback type {state, error} on RMSE_drift_ using two-way, repeated measures ANOVA and post-hoc Tukey *t*-test (where appropriate). For reaching, we analyzed mean absolute error and target capture variability for each target type {center, near, far} by calculating the within-subject differences between performances during the test phase and both the post-training baseline phase and the sham feedback phase. We used paired *t*-test to compare the V_−_T_−_ and V_−_T_+_ (no vibration verses vibration) conditions to determine if state and error feedback encoding could improve performance in the absence of vision. We tested V_−_T_+_ verses V_−_T_sham_ to verify that any performance improvement ascribed to state and/or error feedback was due to the specific information content of the vibrotactile feedback rather the mere presence of vibration. Finally, we used paired *t*-test to compare test phase (V_−_T_+_) performance across feedback conditions {optimal state, error}, to determine which encoding scheme best enhanced performance during reaching to each target type. All statistical testing was carried out within the Minitab computing environment (Minitab, State College, PA). Bonferroni corrections were applied such that effects were considered statistically significant at the α = 0.05 level.

## Results

### Experiment 1 - Optimizing state feedback

**Fig. 5:**
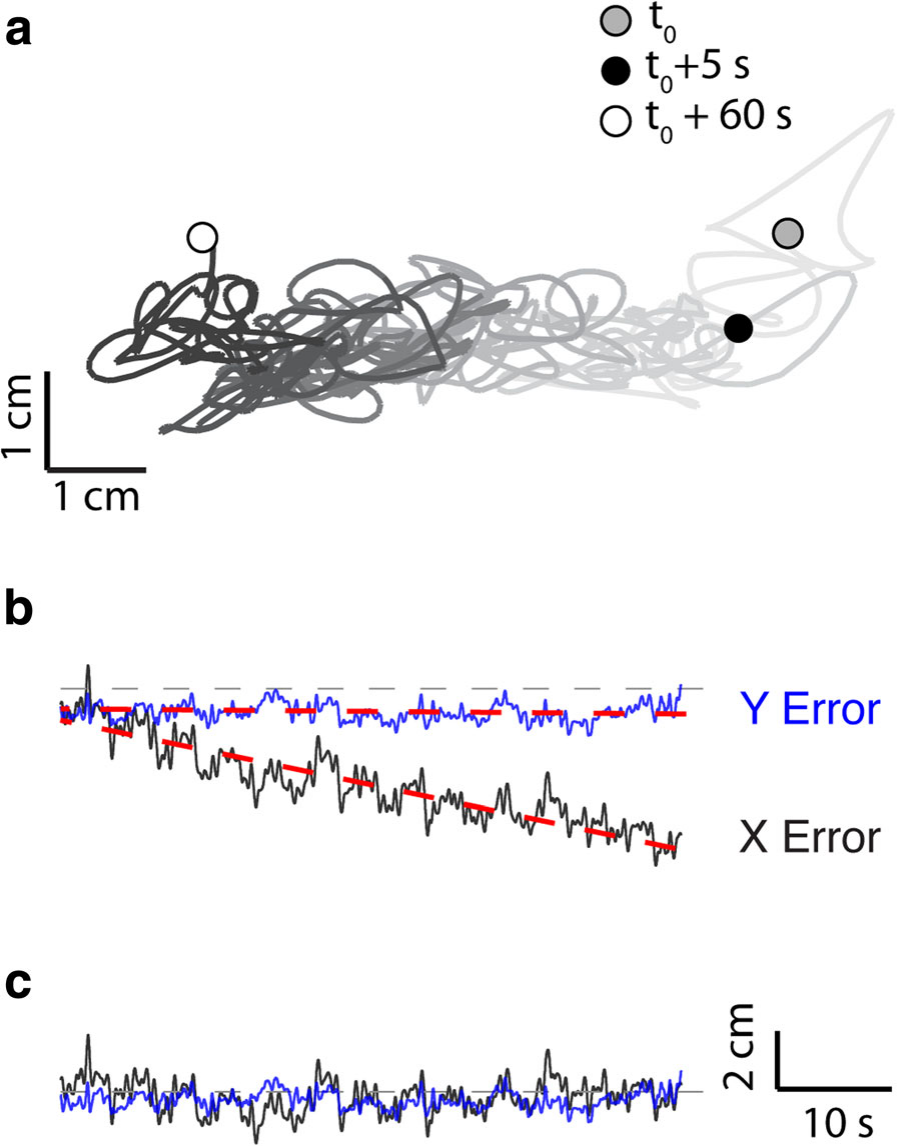
Experiment 1: Selected subject performance in the stabilization task (λ = 1.0). **a** Hand trajectory showing drift over time (*line shading*). Drift was modeled from t = 5 s to the end of the trial at t = 60 s. **b** Time course of the x (*black*) and y (*blue*) components of the endpoint trajectory from t = 5 s to t = 60 s. Scale bars: as in panel **c**. **c** Time course of the x (*black*) and y (*blue*) components of the endpoint trajectory residuals after removal of the drift, from t = 5 s to t = 60 s

Hand position drift was a conspicuous feature of kinematic performance during stabilization in this group of neurologically intact subjects (Fig. 5). As a representative example of this phenomenon, a single trial performed in the absence of visual feedback drifted steadily to the left (Fig. 5a). Drift was well modeled as a linear function of time for both the X- and Y-axis projections (Fig. 5b, red dashed lines). In this way, we decomposed raw hand stabilization kinematics (RMSE_total_) into two parts – components characterized by the linear drift model (RMSE_drift_) and residuals of the modeling process (RMSE_residual_), which reflect the participant’s ability to compensate for moment-by-moment changes in the imposed robotic forces. We observed hand position drift to some degree in all study participants.

**Fig. 6:**
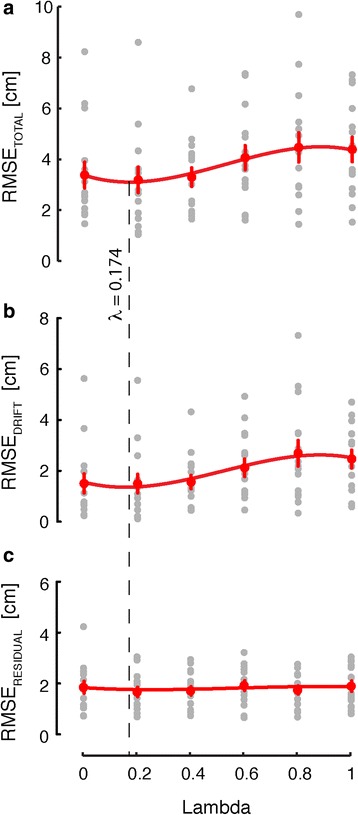
Experiment 1: Population performance in the stabilization task as a function of state mixture parameter lambda, with 3^rd^ order polynomial population fit and 95% function bounds. **a** RMSE of the end-effector trajectory. **b** RMSE of the drift component of the end-effector trajectory. **c** RMSE of the residuals after removal of the drift

Across the study population, RMSE_total_ varied with the state weighting variable (Fig. 6a), with approximately equal contributions of RMSE_drift_ (Fig. 6b) and RMSE_residual_ (Fig. 6c) at low values and an increasing drift contribution at higher values. We fit a third-order polynomial to the pooled population RMSE_TOTAL_ data and found this relationship to be minimized when was approximately 0.2. A similar result was obtained upon fitting a third-order polynomial to the pooled RMSE_drift_ data (Fig. 6b). By contrast, variation across values in the RMSE_residual_ data appeared to be minimal (Fig. 6c).

These observations were confirmed using repeated measures MANOVA to compare stabilization performance {RMSE_total_, RMSE_drift_, RMSE_residual_} across values. MANOVA found significant variation across values [Wilk’s F_(15,188)_ = 2.536; *p* = 0.002]. Subsequent ANOVA found significant variation in RMSE_total_ values [F_(5,70)_ = 6.02, *p* < 0.0005] such that Dunnett multiple comparison tests (referenced to control level: *λ* = 0.2) revealed significant increases in RMSE_total_ when *λ* = 0.6 (*p* = 0.022), *λ* = 0.8 (*p* = 0.001) and *λ* = 1.0 (*p* = 0.001), but not when *λ* = 0.0 or *λ* = 0.4 (*p* > 0.05 in both cases). Similarly, ANOVA [F_(5,70)_ = 5.52, *p* < 0.0005] and Dunnett multiple comparison tests found significant increases in RMSE_drift_ between the control level *λ* = 0.2 and both *λ* = 0.8 (*p* = 0.002) and *λ* = 1.0 (*p* = 0.005). By contrast, ANOVA found no significant variation in RMSE_residual_ across values [F_(5,70)_ = 1.22, *p* = 0.311].

**Fig. 7:**
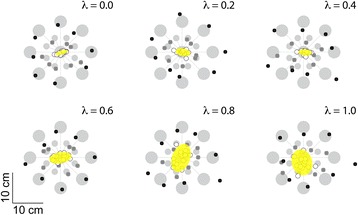
Experiment 1: “Birds-eye view” of selected subject performance in the reaching task for each λ value in V_kr_ visual condition. *Light grey circles*: the 16 targets; *small black dots*: final reach endpoints for movements to the far targets; *small, dark grey dots*: final reach endpoints for movements to the near targets; *white dots*: final reach endpoints for movements returning to the central home target. *Yellow ellipses* represent the two-dimensional 95% confidence intervals of the return-to-home reach endpoints

Kinematic performance of reaching movements also varied with *λ*, even though exposure to each new *λ* encoding scheme was very brief prior to structured reach training. This was most clearly evident in the measure of target capture variability obtained using final hand positions recorded during return-to-home movements. Figure 7 depicts all final hand positions achieved by one subject while reaching in the absence of ongoing visual feedback but in the presence of *λ*- weighted vibrotactile feedback. The yellow ellipses represent 95% CIs on the total distributions of return-to-home movement endpoints recorded under each *λ* feedback condition. The precision of return-to-home reaches degrades substantially with increasing *λ* values. *λ* -dependent variations in performance were more difficult to discern for reaches directed to the near and far targets.

**Fig. 8:**
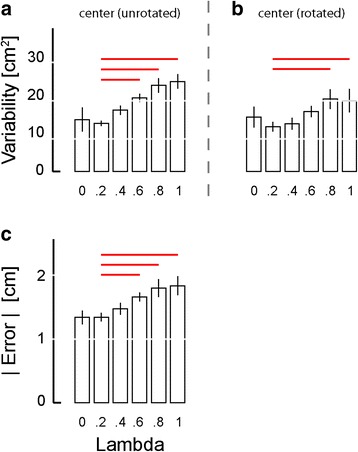
Experiment 1: Population statistics for reaching task, as a function of state mixture parameter lambda. *Error bars* represent ± 1 SEM. **a** Variability of raw reach endpoints about the home target (area of an ellipse fit to the reach endpoints). **b** Variability of reach endpoints at the central target location after collapsing across movement directions. **c** Mean absolute error |Error| at the central target. *Red lines*: significant Dunnett comparisons at *p* < 0.05

Across the study population, target capture variability for return-to-home reaches exhibited significant variation across *λ* values [ANOVA: F_(5,70)_ = 6.01, *p* < 0.0005] such that Dunnett multiple comparison tests revealed significant increases in target capture variability when *λ* = 0.6 (*p* = 0.034), *λ* = 0.8 (*p* = 0.028) and *λ* = 1.0 (*p* = 0.006) when compared to *λ* = 0.2 (Fig. 8a). Increased variability observed when *λ* = 0.0 and *λ* = 0.4 relative to *λ* = 0.2 did not reach statistical significance. This outcome suggests that low values enhance spatial localization of the hand about the central reference location.

We also computed target capture variability and mean absolute error at the center, near and far targets after collapsing across movement directions (i.e., after counter-rotating reach endpoints by the intended movement direction about the home target). MANOVA found significant variation across *λ* values within the six datasets (2 performance measures × 3 target sets) [Wilk’s F_(35,271)_ = 1.986; *p* = 0.001]. Subsequent ANOVA found a significant main effect of *λ* for both performance measures at the center target [F_(5,70)_ > 3.60, *p* < 0.006 in each case] (Figs. 8b, 7c), but no main effect of *λ* for either measure at the near and far target sets [F_(5,70)_ < 1.31, *p* > 0.270 in all cases] (data not shown). At the center target, Dunnett multiple comparison tests revealed significant increases in target capture variability when *λ* = 0.8 (*p* = 0.011) and *λ* = 1.0 (*p* = 0.018) compared to when *λ* = 0.2 (Fig. 8b). Dunnett multiple comparison tests also revealed significant increases in mean absolute error magnitude when *λ* = 0.6 (*p* = 0.025), *λ*= 0.8 (*p* < 0.001) and *λ* = 1.0 (*p* < 0.001) compared to when *λ* = 0.2 (Fig. 8c).

### Experiment 2 - Comparison of optimal state vs. error feedback

**Fig. 9:**
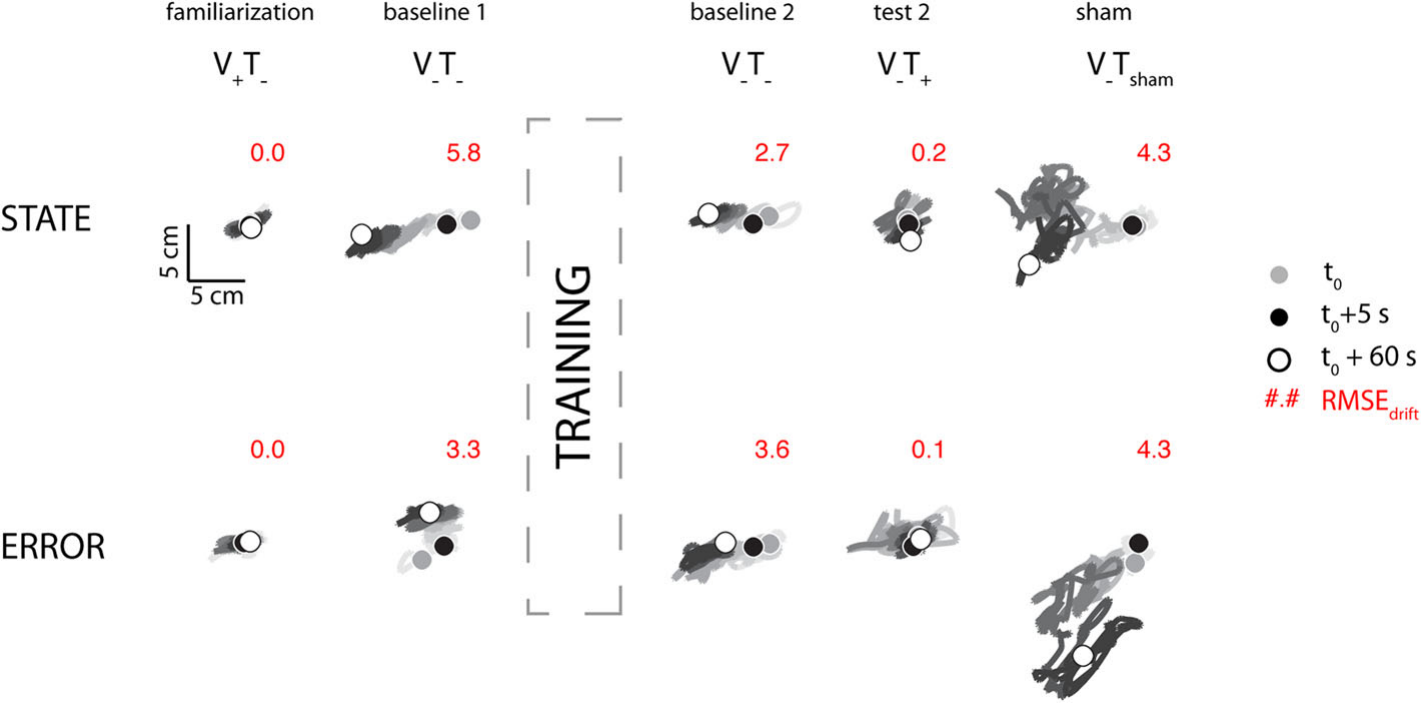
Experiment 2: “Birds-eye view” of selected subject performance in the stabilization task. Cursor trajectory showing drift over time (*line shading*) varies with the presence and type of vibration feedback. *Light grey dot*: hand position at time t_0_; *black dot*: hand position at time t_0_ = 5 s; *white dot*: hand position at time t_0_ = 60s. Drift was modeled from t = 5 s to the end of the trial at t = 60 s. Values in *red* are the RMSE_Drift_ for that trial

Figure 9 contrasts one participant’s stabilization performance during Experiment 2 sessions wherein state feedback (*λ* = 0.2; top) and error feedback (bottom) was administered. During task familiarization trials with ongoing visual feedback but no tactor feedback (V_+_T_−_), the subject readily maintained hand position centered on the home target with virtually no drift. During baseline testing, when visual feedback was subsequently removed and the vibration was not yet introduced (V_−_T_−_), hand position gradually deviated from the home target, albeit in different directions on different trials. After performing about 45 min of training with vibrotactile feedback during reaching and stabilizing, the subject persisted in exhibiting drift during post-training baseline assessment (although drift magnitude appears to have decreased somewhat after training). By contrast, the subject successfully eliminated drift when provided vibrotactile feedback of either state or error feedback (V_−_T_+_). This effect was due to the information contained within the feedback and not the mere presence of vibrotactile stimulation, because the magnitude of drift was at least as great during sham stimulation trials (V_−_T_SHAM_) as it was during the baseline trials.

**Fig. 10:**
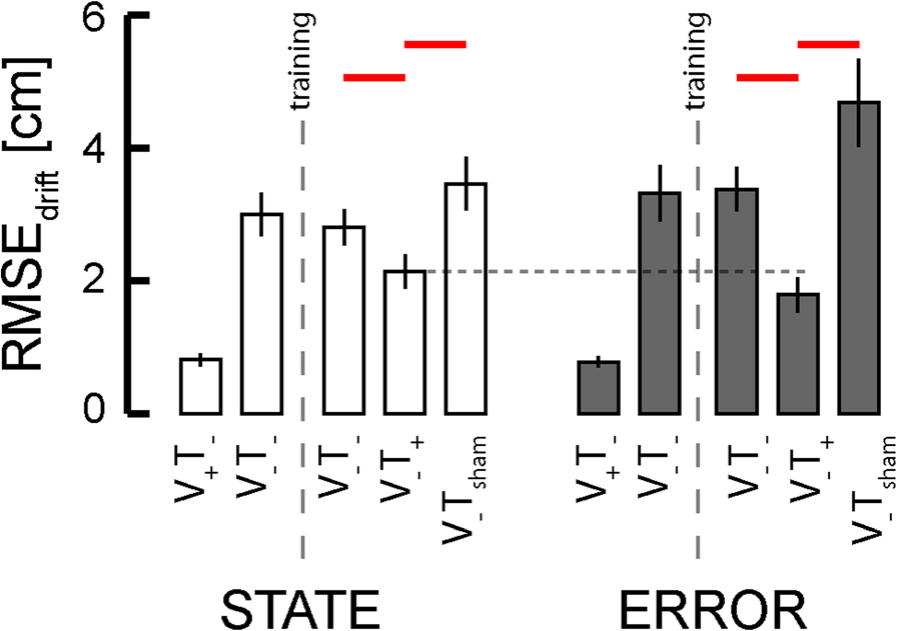
Experiment 2: Population statistics in the stabilization task for error and state feedback. *Red lines*: *p* < 0.05. *Vertical dashed lines* mark the occurrence of training. The *black horizontal dashed line* provides a reference to assist visual comparison across training groups. No significant difference was observed across groups in this condition

Based on these observations, we focused our analysis of population behavior on RMSE_drift_ during the post-training baseline (V_−_T_−_) testing (V_−_T_+_) and sham feedback (V_−_T_SHAM_) phases of this Experiment (Fig. 2c). Two-way repeated measures ANOVA found that when stabilizing about the home target, RMSE_drift_ varied dramatically across experimental phases [F_(2,70)_ = 23.76, *p* < 0.0005] but importantly not across feedback conditions [F_(1,70)_ = 0.56, *p* = 0.457], with no interaction between these two factors [F_(2,70)_ = 2.74, *p* = 0.071] (Fig. 10). Dunnett multiple comparison tests on the effect of experimental phase (control level: V_−_T_+_ test phase) revealed that the significant main effect was the result of a decrease in RMSE_drift_ during the V_−_T_+_ test phase as compared to both the V_−_T_−_ post-training baseline phase (*p* < 0.0005) and the sham stimulation phase (V_−_T_SHAM_: *p* < 0.0005). The difference between the post-training baseline and test phases was not the result of an order effect (i.e., additional practice on the tasks) because the presentation order of these two phases was counter balanced across participants. Moreover, the benefits of vibrotactile feedback were specific to the correction of RMSE_drift_, as separate ANOVA and Dunnetts tests revealed no systematic benefit of test phase vs. post-training baseline performance on RMSE_residual_ values for either feedback type (*p* > 0.211 in both cases).

**Fig. 11:**
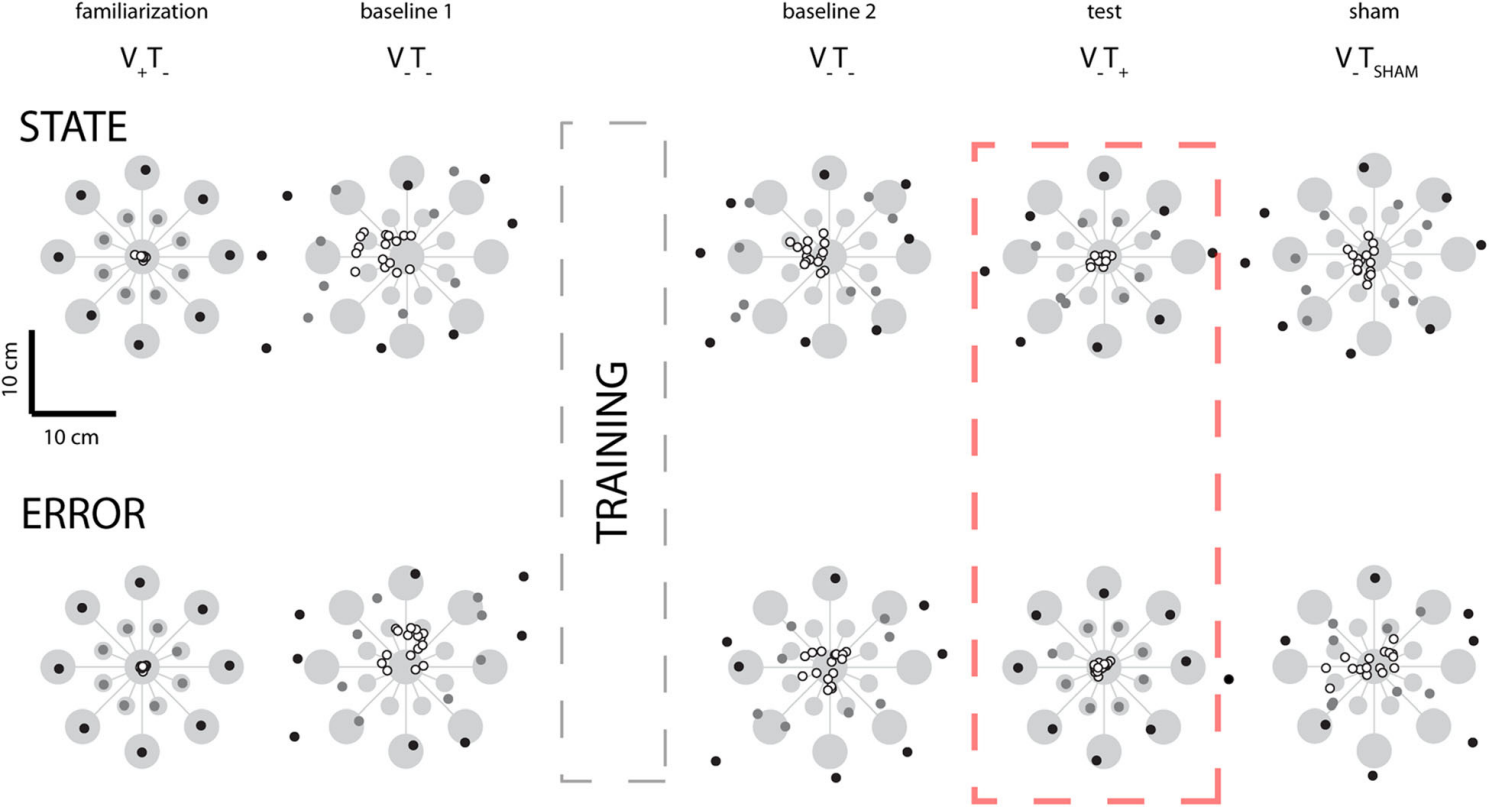
Experiment 2: Selected subject performance in the reaching task. Figure elements as described in the legend for Fig. 8. Compare performance in the test phases (*red dashed box*) to the baseline 2 and sham phases

The absence of a significant main effect of vibrotactile feedback type on RMSE_drift_ during stabilization was not entirely unexpected because, in the neighborhood of the home target, the information content of state and error encoding schemes is really quite similar, deviating by the small amount of velocity information contained within the state feedback. By contrast, the two encoding schemes differ markedly in the neighborhood of targets that are not centered upon the origin of the workspace, defined in this study as the center of the home target. Figure 11 depicts all final hand positions achieved by one subject while reaching in the Experiment 2 sessions wherein state feedback (top) and error feedback (bottom) was administered. During task familiarization trials with ongoing visual feedback but no tactor feedback (V_+_T_−_), the subject captured the center, near and far targets with accuracy and precision. When visual feedback was subsequently removed during pre-training baseline testing (V_−_T_−_), target capture performance collapsed in the sense that reach endpoints deviated wildly from all the intended targets. Reaches to the near and far targets systematically overshot their intended targets whereas the dispersion of return-to-home reaches increased greatly. After performing about 45 min of training with vibrotactile feedback during reaching and stabilizing behaviors, the subject appeared to exhibit beneficial aftereffects of training during the post-training baseline assessment without vibration in the sense that final return-to-home hand positions appeared to cluster more tightly around the center target. Aftereffects of training in this second baseline phase were more difficult to discern at the near and far targets. By contrast, beneficial effects of concurrent vibrotactile feedback were evident and specific to the different encoding schemes during the test phase performed without cursor feedback (V_−_T_+_) (Fig. 11; red dashed box). Although target capture accuracy and precision appeared to improve substantially with concurrent state feedback at all three target sets {center, near, far}, target capture performance with error feedback was undoubtedly superior for reaches to the near and far targets. This striking difference between post-training baseline and test performance was not merely an order-effect, as the presentation order of these two blocks was counter-balanced across subjects. As with stabilization, this beneficial effect of vibrotactile feedback was due to the information contained within the feedback and not the mere presence of vibrotactile stimulation, because the improvements in reach accuracy and precision were eliminated upon switching to sham stimulation (V_−_T_SHAM_).

**Fig. 12:**
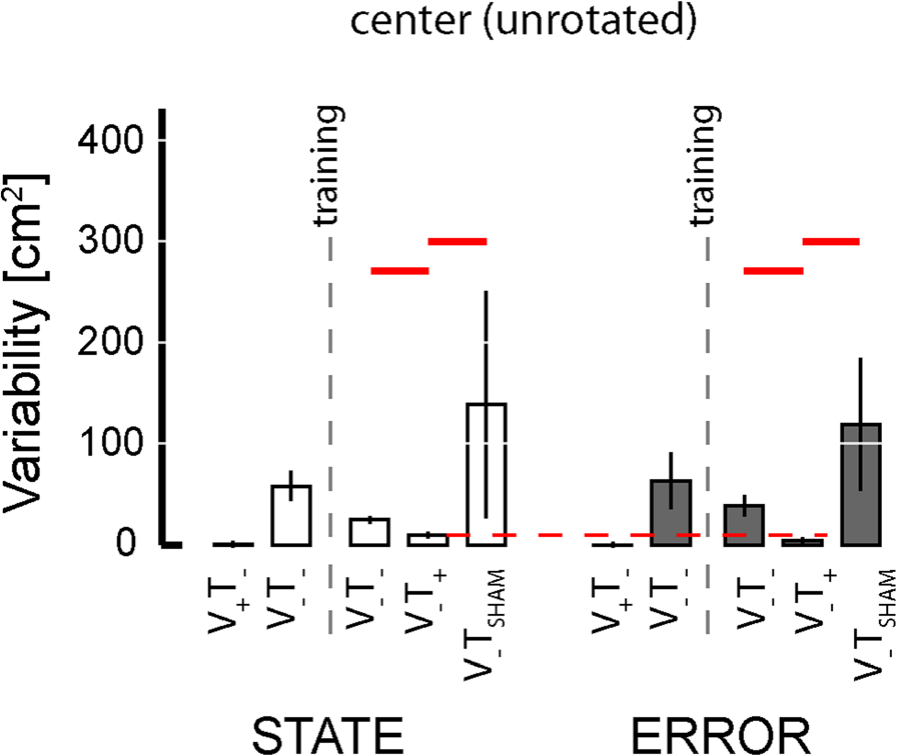
Experiment 2: Population statistics for reaching to the (unrotated) center target. Error bars represent ± 1 SEM. *Red lines*: *p* < 0.05. *Vertical dashed lines* mark the occurrence of training. *Red horizontal dashed line*: significant across-group comparison at *p* < 0.05

We used two-way repeated measures ANOVA to test the ability of optimal state and error feedback schemes to enhance performance of goal-directed return reaches toward the (unrotated) center target (Fig. 12; red significance bars). We found that reach endpoint variability varied systematically across experimental phases [F_(2,70)_ = 42.87, *p* < 0.0005], but not systematically across feedback conditions [F_(1,70)_ = 0.05, *p* = 0.823]. Within both feedback sessions, Dunnett multiple comparison tests revealed that un-rotated center target capture variability in the test block was less than that in the post-training baseline phase (*p* < 0.0005) and in the sham feedback phase (*p* < 0.0005). When we performed the planned comparison of test phase performances across the two feedback encoding schemes (i.e., across experimental sessions), we found that error feedback was better than optimal state feedback in enhancing the precision of return-to-home movements (paired *t*-test: T_14_ = 3.93, *p* = 0.002), with the average ellipse area under state feedback equal to 9.73 cm^2^ and the average ellipse area under error feedback equaling 4.74 cm^2^, with the average within-subject difference equal to 4.99 ± 6.03 cm^2^.

**Fig. 13:**
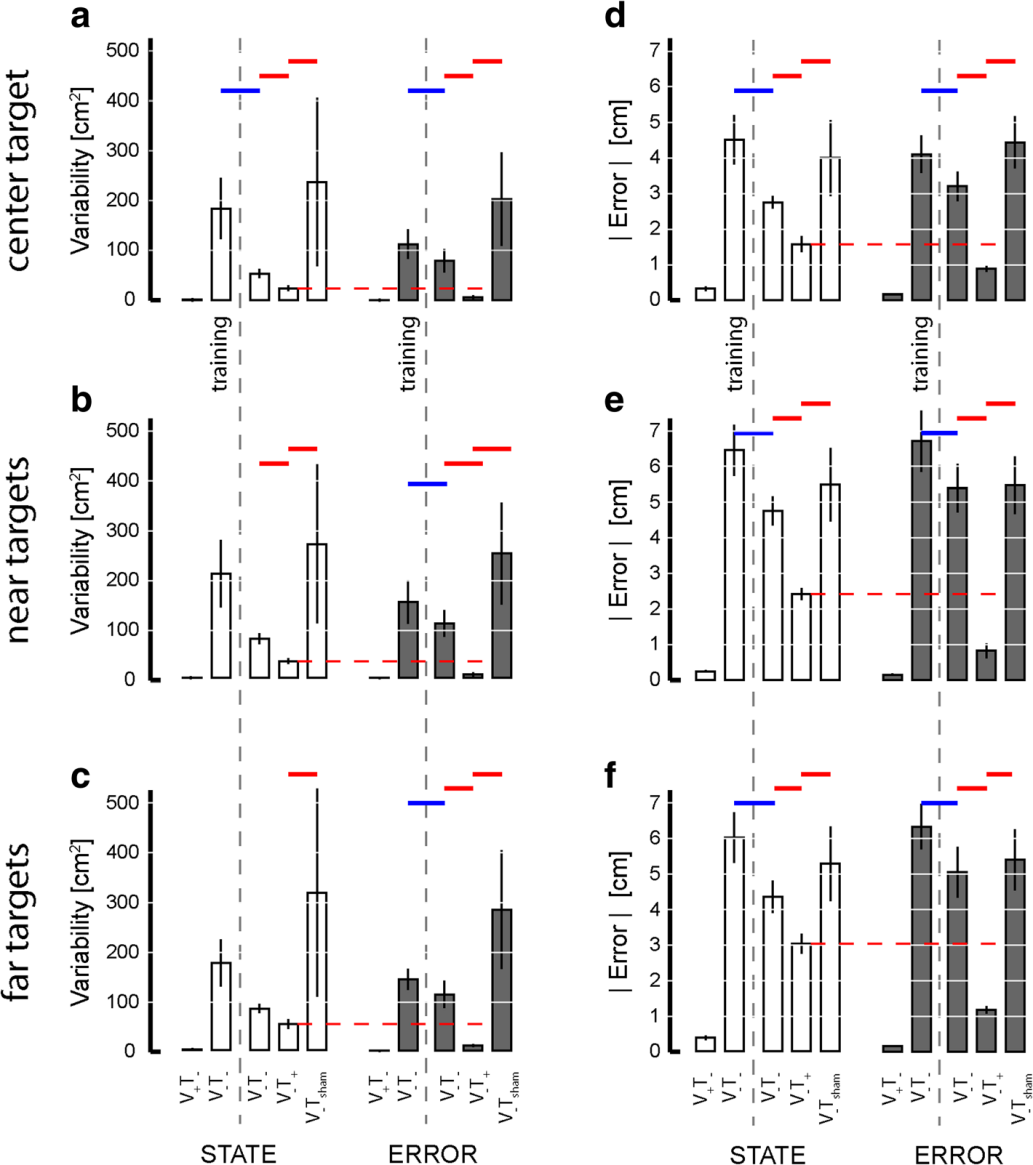
Experiment 2: Population results for reaching task. *Error bars* represent ± 1 SEM. **a**-**c** Variability of reach endpoints for the three target sets after collapsing across movement directions. **d**-**f** Mean absolute error |Error| relative to the center of the target. *Vertical dashed lines* mark the occurrence of training. *Red solid lines*: significant within-group comparisons at *p* < 0.05. *Red horizontal dashed lines*: significant across-group comparisons at *p* < 0.05. *Blue lines*: secondary analysis with *p* < 0.05

Similar outcomes to those presented in Fig. 12 were obtained upon analyzing reaches to all target sets after collapsing across movement directions (Fig. 13; red significance bars). Within both performance measures {reach endpoint variability, mean absolute error magnitude}, MANOVA found significant effects of experimental phase {post-training baseline, test, sham} [Wilk’s F_(4,494)_ = 68.841; *p* < 0.0005], feedback condition {optimal state, error} [Wilk’s F_(2,247)_ = 5.107; *p* = 0.007], and target set {center, near, far} [Wilk’s F_(4,494)_ = 77.362; *p* < 0.0005], as well as a strong interaction between feedback type and experimental phase [Wilk’s F_(4,494)_ = 13.560; *p* < 0.0005]. We therefore performed follow-on ANOVA and post-hoc Dunnett’s multiple comparisons tests to explore this interaction for each of the six combinations of two performance measures {target capture variability, target capture error} and three target sets (Fig. 13).

In contrast to the pattern of performance enhancements observed in Experiment 1, where exposure to each form of vibrotactile feedback encoding was very limited prior to reaching, subjects in Experiment 2 demonstrated the ability to use both forms of feedback to enhance target capture accuracy and precision at all three target sets after approximately 45 min of training. Within the state feedback session, ANOVA found target capture variability to vary significantly across experimental phases at all three target sets [F_(2,44)_ > 8.07, *p* < 0.002 in each case]. Variability was lower in the test block than in the post-training baseline block for nearly all subjects at all three target types, yielding significant and meaningful benefits of vibrotactile feedback at the center (*p* = 0.004) and near targets (*p* = 0.016) (Fig. 13; panels A and B; open bars), with a somewhat more modest trend at the far target (*p* = 0.068) (Fig. 13c; open bars). The benefits of error feedback at all three target types were also very strong [F_(2,44)_ > 43.70, *p* < 0.0005 in each case] (Fig. 13 a-c; filled bars). Post-hoc Dunnett tests found that with error feedback, target capture variability in the test block was less than that in post-training baseline for all target sets (*p* < 0.0005 in each case). The effects for both feedback conditions were specific to the information content embedded within the vibrotactile stimuli because target capture variability in the test phase was less than that in the sham phase for all three target sets under both feedback conditions (*p* < 0.004 in each case). A planned comparison of test phase performance across the two feedback conditions found that error feedback yielded superior reduction in target capture variability at all three targets (paired *t*-test: T_14_ > 4.48, *p* < 0.001 in all three cases).

A similar pattern of results was obtained when we analyzed target capture error within and across feedback sessions (Fig. 13: panels d-f). Comparing within feedback sessions, ANOVA and post-hoc Dunnett tests identified significant reduction in target capture error when informative vibrotactile feedback was provided for each of the three target sets relative to post-training baseline performance (*p* < 0.019 in all six cases). Performance enhancement was specific to the information contained within the vibrotactile feedback because target capture errors during sham vibration far exceeded those during the test phase for all three target sets under both feedback conditions (*p* < 0.001 in each case). A planned comparison of test phase performance across the two feedback conditions found that error feedback yielded superior reduction (vs. state feedback) in target capture error for all three target sets (paired *t*-test: T_14_ > 4.46, *p* < 0.001 in all three cases.).

Although not a main focus of our study, a comparison of target capture performance before and after vibrotactile feedback training provided evidence for a persistent, beneficial effect of vibration training on subsequent reaching movements performed in the absence of both visual and vibrotactile feedback (Fig. 13; blue significance bars). MANOVA found a significant main effects of training [Wilk’s F_(2,160)_ = 9.256; *p* < 0.0005] and target [Wilk’s F_(4,320)_ = 340.761; *p* < 0.0005] on V_−_T_−_ reaching, regardless of feedback condition [Wilk’s F_(2,160)_ = 0.080; *p* = 0.923]. Subsequent ANOVA and Dunnett multiple comparisons tests found strong improvement in reach performance after training with both optimal state (*p* < 0.05 in four of the six cases depicted in Fig. 13) and error feedback (*p* < 0.05 in all 6 cases).

**Fig. 14:**
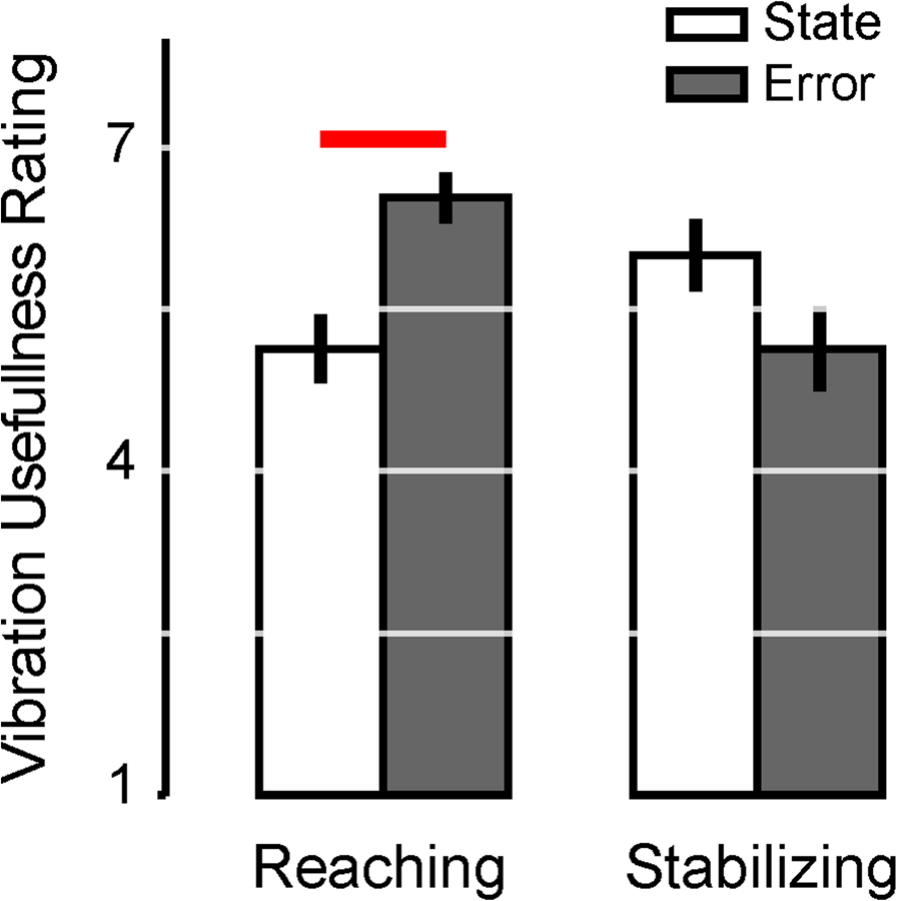
Experiment 2: Assessment of usefulness on a 1–7 scale for state and error feedback for three tasks. *Error bars* represent ± 1 SEM. *Red line*: *p* < 0.05

Finally, survey results suggest that participant preferences were task specific (Fig. 14). During reaching, participants perceived error feedback to be more useful than optimal state feedback (paired *t*-test: T_14_ = 3.42, *p* = 0.004). During stabilization, participants tended to perceive optimal state feedback to be more useful than error feedback, although the statistical significance of this difference did not survive correction for multiple comparisons.

Taken together, the results of this study demonstrate that supplementary vibrotactile feedback can yield both immediate performance enhancements during goal-directed stabilization and reaching actions as well as beneficial aftereffects of training that persist after vibrotactile feedback has been removed.

## Discussion

The ultimate objective of this line of research is to develop sensory substitution technologies that enhance closed-loop control of goal-directed behaviors in people with impaired somatosensation. We sought here to establish the objective and subjective utility of two forms of supplemental vibrotactile feedback – encoding of limb state or hand position error - for enhancing real-time control of arm stabilization and reaching behaviors in unimpaired individuals. To mimic practical constraints experienced by stroke survivors, many of who have lost or impaired somatosensation in their more affected arm, we applied the feedback to a body part not directly involved in the action (i.e., the opposite arm). This approach is reasonable as a first step because most people – even neurologically intact individuals - exhibit imperfect somatosensory control of the arm and hand in the absence of ongoing visual feedback. Moreover, despite the likely differences in etiology of performance deficits in neurologically-intact individuals and those with somatosensory impairment (e.g., hypertonia in the contralesional arm), it also remained to be shown whether it would even be possible to use supplemental vibrotactile feedback applied to one arm to enhance reach and stabilization behaviors performed by the other arm.

Here, in a series of two experiments, we demonstrate for the first time that both vibrotactile encoding schemes can effectively eliminate drift that naturally accrues in the internal representation of hand position [23, 53, 60] in the absence of ongoing visual feedback of hand position. Enahanced kinesthetic control was most evident when subjects stabilized the hand at the origin of the feedback encoding space (i.e., the “home target” for both schemes), even when prior exposure to each particular encoding was limited to just 1 min. Both encoding schemes similarly and immediately promoted accuracy and precision of reaching movements directed toward the origin of the encoding space. However, the two forms of feedback differed in their ability to immediately enhance reach accuracy and precision at other locations in the arms workspace. Whereas 1 min of training did not suffice to allow subjects to use any of the tested forms of state feedback to successfully reach to the near and far target sets in Experiment 1, the best of the state feedback encoding schemes (80% hand position information + 20% hand velocity information) and error feedback both improved reach accuracy and precision at spatial targets throughout the arms reachable workspace after ~45 min of training in Experiment 2. The beneficial effects of state and error feedback were specific to the information content encoded within the vibrotactile stimuli because non-informative sham stimulation failed to elicit any meaningful enhancement of performance in any case. This outcome is important for efforts to develop sensory substitution technologies for neurorehabilitation because it demonstrates that people can learn to use easy-to-implement, opposite-arm state feedback to improve performance of goal-directed stabilization and reaching behaviors in the absence of ongoing visual feedback. Nevertheless, for reaching, error feedback was superior to optimal state feedback, not only on objective measures of target capture accuracy and precision, but also on a subjective measure of perceived utility.

### Importance of information content within supplemental vibrotactile feedback

The model simulations described in Fig. 1 suggest that the control of systems dominated by second-order dynamics with delayed feedback can be enhanced by providing state feedback that adds a modest amount of velocity information to position information (e.g., in a ratio around 20%:80%). The reason for this is that velocity feedback is predictive of position feedback in the sense that the phase of velocity information at any given frequency leads position information by 90° and may therefore enhance perception of changes in limb state. Experiment 1 was designed to test the model predictions. Although we observed minimization of hand position drift during stabilization (Fig. 6) and optimization of reach accuracy and precision at the center target (Fig. 8) when vibrotactile feedback mostly encoded position feedback, we did not observe significant variations in the ability of participants to reject moment-by-moment fluctuations in robotic force perturbations when we systematically varied the composition of state feedback. We suspect this negative outcome was due to the limited amount of training with each different combination of state feedback in the first experiment. Indeed, a comparison of reach performance at the near and far targets across both experiments suggests that the cohort of individuals tested here required up to 45 min of training with the optimal state encoding scheme to begin to learn how to use that form of feedback. Because training-dependent performance enhancements were limited to drift reduction and the targeting of point-to-point reaches (i.e., we observed no significant improvement in RMSE_residual_), it is likely that full integration of supplemental kinesthetic feedback into the moment-by-moment control of the arm (e.g., during stabilization) requires considerably more training than the mere moments (Experiment 1) or minutes (Experiment 2) provided in the current study, and/or training more directly focused on the stabilization task (rather than on reaching as in the current study).

Nevertheless, our data demonstrate that it is indeed possible to achieve usable sensory substitution without compensating for nonlinearities in the relationship between vibrotactile stimulus intensity and perception (Verrillo et al., 1970; 1972), or for nonlinearities introduced by the tactors themselves (Fig. 3b). A likely reason for this outcome is the “soft” nature of the nonlinearities described both by Verrillo and colleagues and by Fig. 3b (no sharp discontinuity is present in the stimulus-to-percept mapping). Based on our observations and participant comments, it is furthermore likely that conscious perception of vibrotactile stimuli can vary somewhat from day to day, and even perhaps within a single experimental session; this did not preclude the ability of participants to use the feedback effectively even though we did not attempt to compensate for such variations.

We chose not to include velocity information in our error encoding scheme and considered a simpler form of goal-aware feedback involving only position information. Including error velocity feedback would not change the “goal-aware” nature of the supplemental feedback. As mentioned in the Background, we have already demonstrated the efficacy of a sophisticated goal-aware encoding scheme that uses an optimal control model as a “teacher” to encode position and velocity error information within the vibrotactile information stream [58]. Here we have shown that even one of the simplest forms of goal-aware encoding out-performs the optimized state feedback encoding when tested after short-term task training. This outcome is unlikely to change for any other optimized goal-aware encoding scheme.

The subjective feedback provided by participants after experiencing each form of state feedback in Experiment 1 was enlightening. During pilot testing, we observed that many participants attempted to solve the stabilization task by stiffening the arm rather than by using the vibrotactile feedback as we had intended them to do. We therefore developed task instructions (and repeatedly reminded the subjects) to avoid stiffening the arm and to use the vibration to accomplish each task. In response to investigator queries about the utility of each mixture of state feedback during the survey period after each trial, participants reported that they did in fact use the vibration as instructed in most cases. However, when the vibration contained more velocity information than position information, some participants reported that they had to temporarily stiffen their moving arm in order to discern the position information within the vibration signal so that they could then make effective use of it. Thus, depending on the value of, different state feedback encodings yielded strikingly different subjective experiences that could elicit different strategies for integrating the supplemental feedback into real-time control of the arm. This is perhaps to be expected because the human brain naturally and fluidly switches between impedance control, feedforward control, and real-time feedback control depending on environmental context and the amount of training within that context [17, 44, 54]. To characterize the integration of supplemental vibrotactile feedback of kinesthetic performance into ongoing sensorimotor control, future efforts may wish to quantify the occurrence of arm-stiffening (via EMG or other means) and the relative ratio of impedance-to-feedback control as that ratio evolves during long-term training with the vibrotactile display system.

Qualitative differences in the nature of state and error feedback encodings can help explain performance differences in reach to the near and far targets in Experiment 2. Because we defined the origin of the arm’s workspace to be at the center of the home target, error and state feedback encodings were similar when was small (e.g., 0.2) but statistically independent when = 1.0. By contrast, the origin of the error encoding scheme jumps to the current goal location, wherever it happens to be within the workspace. Thus, when the target jumps to a far target, the origin of error encoding jumps to that location whereas the origin of state encoding never changes. Thus, when driven by error feedback, test phase target capture performance at the near and far targets in Fig. 11 closely replicated performance at the home target in that same test phase, and resembled performance at all targets driven by visual feedback in the familiarization phase. But whereas test phase performance driven by optimal state feedback was far better than the baseline and sham phases, target capture variability and RMS error at all target sets was about twice as great with optimal state encoding compared to that observed with error encoding.

The qualitative differences between state and error feedback encoding are also important from the perspective of implementing a practical, supplemental feedback delivery system. Whereas it is easy to implement an error encoding scheme during highly constrained, lab-based stabilization and reaching tasks that utilize a robotic manipulandum to measure instantaneous hand position relative to well-defined spatial targets, it will be much more difficult to define error feedback using wearable technology that must predict the intent and movement goals of the user on a moment-by-moment basis in a unstructured, real-world environment. Although we are not currently aware of technology that is competent to perform intent and goal prediction in uncertain environments, low-cost wearable technologies currently integrate MEMS accelerometers, gyroscopes and magnetometers and can be used to estimate limb state. We do not, however, believe intent and goal prediction to be insurmountable hurdles, as real-time computing systems already are capable of providing goal-aware feedback encodings that enhance human performance of difficult but well-defined tasks such as balancing an inverted pendulum while minimizing both kinematic error and control effort [58].

### Exposure to vibrotactile feedback of limb state induces spatial learning

The tasks subjects performed in the current experiments required them to learn how hand position in the horizontal plane mapped onto target locations within the vertical plane of the display screen. More specifically, when subjects reached to visual targets, they needed to learn an inverse kinematic map specifying how a desired change in visual cursor position should map onto an appropriate, desired change in hand position. By requiring subjects to perform the experimental tasks using vibrotactile cues, we required them to learn at least two features of an additional, interposed transformation: 1) how hand motions influence activity within the vibrotactile display; and 2) whether and how changes in visual target location modulate the patterns of activity within the vibrotactile display. Experiment 1 probed the first of these questions and provided evidence that a state encoding with = 0.2 enhanced stabilization and return-to-home reaches better than several other state encoding schemes tested. Experiment 2 probed the second question and provided evidence that within the time frame of a single experimental session, error feedback out-performed optimal state feedback in facilitating reaching, particularly to the near and far targets.

Importantly, two experimental observations provide evidence that state feedback did encourage subjects to learn this additional spatial map, thus providing a sound rationale for further development of state feedback-based supplemental feedback systems. First, after ~45 min of practice with optimal state feedback, we observed performance improvements at the near and far targets during test phase reaching with vibrotactile feedback. Because the order of V_−_T_+_ test and V_−_T_−_ baseline phases after training was counter-balanced across subjects, we can reject the possibility that this learning effect was due to more prolonged practice on the reaching task in the V_−_T_+_ condition. We conclude therefore that subjects used the optimal state feedback to improve accuracy and precision of their reaches. Because the near and far targets mapped onto non-zero activation patterns in the vibrotactile display, the observed performance enhancements were not confounded by enhancements that might be due to any similarity with error encoding (e.g., that which exists at the center target). Second, we observed less overshoot in post-training baseline reaches performed without ongoing vibrotactile stimulation (relative to pre-training baseline), especially at the near targets. This observation suggested that prolonged training with optimal state vibrotactile feedback facilitated learning of an internal representation or map of space that was subsequently recalled during post-training baseline testing to guide reaches to visual targets. Because we saw evidence of this second aspect of spatial learning at all three target sets with state feedback, especially with regards to target capture error (RMSE) (Fig. 13, blue significance bars), it is possible that optimal state feedback can be as effective as error feedback at encouraging subjects to learn the spatial relationships between target location, hand position and vibrotactile stimulation. Future studies should be designed and conducted specifically to explore how subjects learn to use supplemental vibrotactile feedback to shape internal representations of body configurations.

### Potential applications of supplemental vibrotactile stimulation

Wearable technologies designed to augment human motor performance by providing supplemental vibrotactile stimulation have many potential applications. In perhaps its simplest form, stochastic resonance (c.f., [62]), vibrotactile stimulation can enhance behaviors such as standing balance [38] and grip force production [16] via application of subsensory, random, vibrotactile signals onto the soles of the feet or onto the tendon of finger flexor muscles. Stochastic resonance is a nonlinear, cooperative effect in which a weak stimulus of interest (e.g., forces applied to cutaneous mechanoreceptors) entrains large-scale environmental fluctuations (e.g., the injected noise), with the result that the sensitivity of a nonlinear threshold stimulus detector (e.g., cutaneous mechanoreceptor) is greatly enhanced [62]. In our study, the beneficial effects of vibrotactile stimulation are not the result of stochastic resonance because performance improvements dissipated in the presence of sham stimulation.

A recent set of experimental studies has found that artificial activation of sensory afferents of distal arm musculature (i.e., at the wrist) can improve control of reaching, stabilizing and tracking behaviors performed with the proximal arm (i.e., shoulder and elbow) by adding excitatory drive to central and peripheral sensorimotor control structures ([12, 13]; b). For example, Conrad and colleagues [14] applied unmodulated, 70 Hz wrist tendon vibration on the paretic arm of 10 stroke survivors as they made planar, center-out arm movements. Relative to performances measured before the onset of vibrotactile stimulation, three aspects of performance were enhanced during stimulation and for a brief period after stimulation had ceased: hand position stability at the end of reach improved; muscle activity throughout the arm decreased, and grip pressure during movement decreased. As discussed in [13], possible mechanisms behind improved proximal arm control in response to distal wrist tendon vibration may include improved central (i.e., cortical) sensorimotor integration within spared neural circuits already mediating control of the hemiparetic arm or improved cortical modulation of spinal reflex activity, which acts to elevate spinal reflex thresholds (thereby reducing spastic hypertonia). The beneficial effects of vibrotactile stimulation in our study do not share a common mechanism of action with the effects studied by Conrad and colleagues. Whereas Conrad and colleagues provided vibrotactile feedback that did not itself encode any meaningful information, the effects of our vibrotactile stimulation were specific to the type of information encoded within the tactile data stream.

Many research teams have proposed using vibrotactile displays to inject useful information into the human nervous system. Several recent application include the use of tactile navigation displays for aircraft pilots seeking to fly toward a target [59], to hover a helicopter [40], to provide mission critical information such as which direction is down during conditions of low visibility [51], and to enable vibrotactile transmission of spoken language [34]. By injecting informative vibrotactile feedback to the non-moving arm in our study, we are recruiting alternate sensorimotor control pathways into the task of controlling the moving arm. The results of the present study show that doing so for neurologically intact people can improve the accuracy of goal-directed reaching in the absence of ongoing visual feedback, and eliminate limb position drift during limb stabilization without visual feedback. Doing so for stroke survivors who retain some motor strength and the capacity to produce flexion and extension torques in the proximal involved arm could promote increased use of that arm by allowing them once again to “feel” movement, thus enhancing arm control in the absence of visual feedback. Future studies should explore the limits of this approach to vibrotactile sensory substitution in this population by quantifying effective bandwidth of control with and without vibrotactile feedback, relative to the bandwidth of control attainable using visual feedback alone.

Although a focus on neural mechanisms is beyond the scope of the current study, we note that vibration and joint position sense both follow the dorsal column/medial lemniscus system that projects through the ventral posterior lateral nucleus of the thalamus to primary sensory cortex. As shown via functional neuroimaging [46, 53], these brain regions contribute importantly to the real-time, closed-loop control of the distal upper extremity. They are also susceptible to injury from the most common form of stroke. Recent studies reveal networks of neurons interconnecting two sides of the gray matter at the brainstem and spinal levels as well as intrahemispheric transcallosal connections that may form “detour circuits” for recovery of function (for review, see [22]). We therefore speculate that “detour circuits” may provide a way for the supplemental kinesthetic feedback we describe to tap into residual cerebello-thalamo-cortical circuits that participate in the real-time, closed-loop control of the contralesional arm and hand. Results from a pilot study involving a small cohort of stroke survivors demonstrates that some survivors can indeed use vibrotactile feedback applied to the ipsilesional arm to improve control of the contralesional arm, thereby suggesting that our general approach may have utility in this population [57]. However, it remains to be determined whether the specific “optimal” combination of position and velocity feedback identified here as optimizing RMS error and target capture variability in neurologically intact individuals will generalize to stroke patients, or to other tasks such as pursuit tracking of a moving target.

Subjects in the current study perceived error feedback to be more useful than state feedback during the reaching task, but exhibited no clear preference for either form of feedback while stabilizing about the home target. This outcome makes sense because state and error feedback were virtually identical at the home target in both tasks. Either spontaneously or during the survey period at the end of the session, every participant in Experiment 2 reported that they preferred the presence of informative vibration during training and test phases over no vibration. Many participants expressed dismay or frustration when asked to repeat the task without vibration after they had been able to practice with vibration. These subjective results reflect positively on the user experience of wearable technologies using vibrotactile encoding of state and/or error feedback for the purpose of enhancing motor performance of goal-directed actions with the arm.

A limitation of our approach is that we were unable to identify in all participants a common configuration of the vibrotactile display that would allow effective discrimination between activations within all tactor pairs. Instead, tactor placements had to be individualized in 16 of the 26 participants. Even though these 16 participants could initially feel each tactor when individually activated, they reported that for some pairs, they only felt one tactor vibrating even when both were turned on. The most common difficulty was interference between the upper arm and forearm tactors. Adjusting the upper arm tactor slightly to the right or to the left usually resolved this issue. In some cases the forearm tactor instead had to be shifted. One participant did not complete the study and was replaced because she could not discriminate tactor vibrations despite multiple tactor adjustments. We suspect that these difficulties were attributable, in part, to normal variations in the distribution of dermatome innervations across individuals [29]. Another common difficulty was that the internal forearm tactor felt dull. This was typically resolved by shifting the tactor around the muscle or distally towards the wrist. Some participants reported the hand tactor felt much stronger than the other tactors. This perception was reduced by moving the hand tactor towards the wrist. For most participants, adjusting the tactor(s) by 1 to 2 cm was enough to fix the problem. Preliminary psychophysical studies using the same tactors as in the present study suggest that there are systematic differences in vibration perception (discriminability) across dermatomes in healthy human subjects [47, 48]. However, in the current study, a few participants demonstrated variability in vibrotactile perception between sessions such that tactor placement needed to be adjusted from one session to the next. Future work should explore the impact of location-dependent variations in vibrotactile perception and control, and we recommend future applications to attend carefully to this source of variability.

A second limitation of our study is that we do not know the extent to which the frequency content of our robotic perturbations may have exceeded the effective closed-loop bandwidth of control using supplemental vibrotactile feedback. The relative insensitivity of RMSE_RESIDUAL_ errors – but not RMSE_DRIFT_ – to the different λ values (Fig. 6c) suggests this may have been the case. A future study could, for example, use a chirp-stimulus, rotary-pursuit tracking task to identify the closed-loop bandwidth of control for each of several different forms of supplemental vibrotactile feedback. We anticipate that a pursuit task will be much more amenable to frequency domain analysis than stabilizing against deterministic robotic perturbations and point-to-point reaching as studied here, and therefore more informative about frequency limitations of supplemental vibrotactile feedback control. Care will need to be taken when designing the tracking stimulus, however, because we have previously found that neurologically-intact people can readily anticipate and compensate for deterministic, low-frequency force perturbations (e.g., a 6 N force vector rotating in the horizontal plane at 0.25 Hz), but struggle to compensate for much weaker and more complex high-frequency sum-of-sinusoids force perturbations [32], such as those employed in the current study.

## Conclusions

The results of this study have established the immediate utility and relative merits of two forms of vibrotactile kinesthetic feedback in enhancing stabilization and reaching actions performed with the arm and hand in neurotypical people. Whereas the first set of experiments identified one specific combination of hand position and velocity information that optimized state feedback control of stabilization and reaching actions after very limited practice, the second set found that error feedback – a simple form of “goal-aware feedback” - yielded superior performance relative to optimized state feedback throughout the reachable workspace. These results are important because they demonstrate that the intact human brain is capable of integrating vibrotactile kinesthetic feedback into the ongoing control of the moving arm and hand, even when that feedback is applied to a body part not directly involved in the action (i.e., the other arm). These findings provide strong empirical evidence motivating and guiding future development of sensory substitution technologies seeking to counteract impaired proprioceptive sensation in stroke survivors who retain motor capacity in the more affected arm.

## Additional file

Additional file 1: Experimental data. (XLSX 30 kb)

## Abbreviations

ANOVA: Analysis of variance
API: Application programming interface
CI: Confidence interval
FSR: Full scale range
Hz: Hertz
KR: Knowledge of results
m: Meter
MANOVA: Multivariate analysis of variance
N: Newton
RMSE: Root mean squared error
V_+_T_−_: Visual feedback and no vibrotactile feedback
V_KR_T_−_: Visual knowledge of results and no vibrotactile feedback
V_−_T_−_: No visual feedback and no vibrotactile feedback
V_−_T_+_: No visual feedback and informative vibrotactile feedback
V_−_T_SHAM_: No visual feedback and sham vibrotactile feedback

## References

[1] Abela E, Missimer J, Wiest R, Federspiel A, Hess C, Sturzenegger M, Weder B (2012). Lesions to primary sensory and posterior parietal cortices impair recovery from hand paresis after stroke. PLoS One.

[2] Badke MB, Sherman J, Boyne P, Page S, Dunning K (2011). Tongue-based biofeedback for balance in stroke: results of an 8-week pilot study. Arch Phys Med Rehabil.

[3] Bark K, Khanna P, Irwin R, Kapur P, Jax SA, Buxbaum LJ, Kuchenbecker KJ. Lessons in using vibrotactile feedback to guide fast arm motions. In: Proc IEEE World Haptics Conference; 2011. p. 355–360.

[4] Bark K, Hyman E, Tan F, Cha E, Jax SA, Buxbaum LJ, Kuchenbecker KJ (2015). Effects of vibrotactile feedback on human learning of arm motions. IEEE Trans Neural Syst Rehabil Eng.

[5] Bastian HC (1887). On different kinds of aphasia, with special reference to their classification and ultimate pathology. Br Med J.

[6] Blennerhassett JM, Matyas TA, Carey LM (2007). Impaired discrimination of surface friction contributes to pinch grip deficit after stroke. Neurorehabil Neural Repair.

[7] Box GEP, Cox DR (1964). An analysis of transformations. J R Stat Soc Ser B.

[8] Cameron BD, de la Malla C, Lopez-Moliner J (2014). The role of differential delays in integrating transient visual and proprioceptive information. Front Psychol.

[9] Carey L, Matyas T (2011). Frequency of discriminative sensory loss in the hand after stroke in a rehabilitation setting. J Rehabil Med.

[10] Casadio M, Sanguineti V, Morasso PG, Arrichiello V (2006). Braccio di Ferro: a new haptic workstation for neuromotor rehabilitation. Technol Health Care.

[11] Connell LA, Lincoln NB, Radford KA (2008). Somatosensory impairment after stroke: frequency of different deficits and their recovery. Clin Rehabil.

[12] Conrad MO, Gadhoke B, Scheidt RA, Schmit BD (2015). Effect of tendon vibration on hemiparetic arm stability in unstable workspaces. PLoS One.

[13] Conrad MO, Scheidt RA, Schmit BD (2011). Effects of wrist tendon vibration on arm tracking in people poststroke. J Neurophysiol.

[14] Conrad MO, Scheidt RA, Schmit BD (2011). Effects of wrist tendon vibration on targeted upper-arm movements in poststroke hemiparesis. Neurorehabil Neural Repair.

[15] Dukelow SP, Herter TM, Moore KD, Demers MJ, Glasgow JI, Bagg SD, Norman KE, Scott SH (2010). Quantitative assessment of limb position sense following stroke. Neurorehabil Neural Repair.

[16] Enders LR, Hur P, Johnson MJ, Seo NJ (2013). Remote vibrotactile noise improves light touch sensation in stroke survivors’ fingertips via stochastic resonance. J Neuroeng Rehabil.

[17] Franklin DW, Osu R, Burdet E, Kawato M, Milner TE (2003). Adaptation to stable and unstable dynamics achieved by combined impedance control and inverse dynamics model. J Neurophysiology.

[18] Ghez C, Gordon J, Ghilardi MF (1995). Impairments of reaching movements in patients without proprioception. II Effects of visual information on accuracy. J Neurophysiol.

[19] Grillner S. Supraspinal and segmental control of static and dynamic gamma-motoneurones in the cat. Goeteborg. Acta Physiol Scand Suppl. 1969;327:1-34. 4313461

[20] Heenan M, Scheidt RA, Woo D, Beardsley SA (2014). Intention tremor and deficits of sensory feedback control in multiple sclerosis: a pilot study. J Neuroeng Rehabil.

[21] Houk J, and Rymer W. Neural control of muscle length and tension. *Comprehensive Physiology.* 1981.

[22] Jankowska E, Edgley SA (2006). How can corticospinal tract neurons contribute to ipsilateral movements? A question with implications for recovery of motor functions. Neuroscientist.

[23] Jeannerod M (1989). The neural and behavioural organisation of goal directed movements.

[24] Johnson RA, and Wichern DW. Applied multivariate statistical analysis. Englewood Cliffs, NJ: Prentice-Hall, Inc; 1988.

[25] Kaczmarek K, Webster J, Bach-Y-Rita P, Tompkins W (1991). Electrotactile and vibrotactile displays for sensory substitution systems. IEEE Trans Biomed Eng.

[26] Krueger A, Giannoni P, Casadio M, and Scheidt R. Optimizing supplemental vibrotactile feedbacl for real-time control of arm stabilization behaviors in humans. In: American Conference on Human Vibration. Milwaukee WI.: 2016.

[27] Lee B, Chen S, Sienko K (2011). A wearable device for real-time motion error detection and vibrotactile instructional cuing. IEEE Trans Neural Syst Rehabil Eng.

[28] Lee B, Kim J, Chen S, and Sienko K. Cell phone based balance trainer. J Neuroeng Rehabil. 9. 2012;9:10. 10.1186/1743-0003-9-10 PMC3340298 22316167

[29] Lee M, Mcphee R, Stringer M (2008). An evidence-based approach to human dermatomes. Clin Anat.

[30] Lieberman J, Breazeal C (2007). TIKL: development of a wearable bibrotactile feedback suit for improved human motor learning. IEEE Trans Robot.

[31] Loeb G (1990). Cochlear prosthetics. Annu Rev Neurosci.

[32] Mrotek LA, Stoeckmann T, Bengtson M, Ghez C, Scheidt RA. Deficits of sensorimotor control and their impact on limb stabilization post-stroke: a case series. Am Soc Neur Rehab, San Diego, CA. 2013.

[33] Nolan MF (1982). Two-point discrimination assessment in the upper limb in young adult men and women. Phys Ther.

[34] Novich SD, Eagleman DM (2015). Using space and time to encode vibrotactile information: toward an estimate of the skin’s achievable throughput. Exp Brain Res.

[35] Oliveira LF, Simpson DM, Nadal J (1996). Calculation of area of stabilometric signals using principal component analysis. Physiol Meas.

[36] Paillard J, Brouchon M, Freedman SJ (1968). Active and passive movements in the calibration of position sense. The neuro-psychology of spatially oriented behavior.

[37] Peterka R, Wall C, Kentala E (2006). Determining the effectiveness of a vibrotactile balance prosthesis. J Vestibular Research.

[38] Priplata AA, Niemi JB, Harry JD, Lipsitz LA, Collins JJ (2003). Vibrating insoles and balance control in elderly people. Lancet.

[39] Proske U, Gandevia SC (2012). The proprioceptive senses: their roles in signaling body shape, body position and movement, and muscle force. Physiol Rev.

[40] Raj AK, Kass SJ, Perry JF (2000). Vibrotactile displays for improving spatial awareness.

[41] Sainburg R, Poizner H, Ghez C (1993). Loss of proprioception produces deficits in interjoint coordination. J Neurophysiol.

[42] Sarlegna F, Gauthier GM, Bourdin C, Vercher JL, Blouin J (2006). Internally driven control of reaching movements: a study on a proprioceptively deafferented subject. Brain Res Bull.

[43] Scheidt RA, Conditt MA, Secco EL, Mussa-Ivaldi FA (2005). Interaction of visual and proprioceptive feedback during adaptation of human reaching movements. J Neurophysiol.

[44] Scheidt RA, Ghez C, Asnani S (2011). Patterns of hypermetria and terminal cocontraction during point-to-point movements demonstrate independent action of trajectory and postural controllers. J Neurophysiol.

[45] Scheidt RA, Stoeckmann T (2007). Reach adaptation and final position control amid environmental uncertainty after stroke. J Neurophysiol.

[46] Scheidt RA, Zimbelman JL, Salowitz NM, Suminski AJ, Mosier KM, Houk J, Simo L (2012). Remembering forward: neural correlates of memory and prediction in human motor adaptation. Neuroimage.

[47] Shah V, Gagas M, Krueger A, Iandola R, Casadio M, and Scheidt R. Vibrotactile discrimination in the upper extremity of healthy human subjects. In: American Conference on Human Vibration. Milwaukee WI: 2016.

[48] Shah V, Gagas M, Krueger A, Iandolo R, Peters D, Casadio M, Scheidt R (2016). Vibrotactile discrimination thresholds vary among dermatomes in the upper extremity of healthy humans.

[49] Shull PB, Damian DD. Haptic wearables as sensory replacement, sensory augmentation and trainer – a review. J Neuroeng Rehabil. 2015; 12:59. 10.1186/s12984-015-0055-z PMC4506766 26188929

[50] Sienko K, Balkwill M, Oddsson L, Wall C (2008). Effects of multi-directional vibrotactile feedback on vestibular-deficient postural performance during continuous multi-directional support surface perturbations. J Vestibular Research.

[51] Sklar A, Sarter N (1999). Good vibrations: tactile feedback in sup- port of attention allocation and human-automation coordination in event-driven domains. Hum Factors.

[52] Smeets JB, Dobbelsteen JJ, Grave DD, Beers RJ, Brenner E (2006). Sensory integration does not lead to sensory calibration. Proc Natl Acad Sci.

[53] Suminski AJ, Rao SM, Mosier KM, Scheidt RA (2007). Neural and electromyographic correlates of wrist posture control. J Neurophysiol.

[54] Takahashi CD, Scheidt RA, Reinkensmeyer DJ (2001). Impedance control and internal model formation when reaching in a randomly varying dynamical environment. J Neurophysiol.

[55] Taub E, Miller NE, Novack TA, Cook EW, Fleming WC, Nepomuceno CS, Connell JS, Crago JE (1993). Technique to improve chronic motor deficit after stroke. Arch Phys Med Rehabil.

[56] Tyson SF, Hanley M, Chillala J, Selley AB, Tallis RC (2008). Sensory loss in hospital-admitted people with stroke: characteristics, associated factors, and relationship with function. Neurorehabil Neural Repair.

[57] Tzorakoleftherakis E, Bengtson MC, Mussa-Ivaldi FA, Scheidt RA, and Murphey TD. Tactile proprioceptive input in robotic rehabilitation after stroke. Conf Proc IEEE ICRA Soc: Seattle; 2015.

[58] Tzorakoleftherakis E, Murphey TD, and Scheidt RA. Augmenting sensorimotor control using “goal-aware” vibrotactile stimulation during reaching and manipulation behaviors. Exp Brain Res. 2016. 10.1007/s00221-016-4645-1 27074942

[59] Van Erp J (2005). Presenting directions with a vibrotactile torso display. Ergonomics.

[60] Wann JP, Ibrahim SF (1992). Does limb proprioception drift?. Exp Brain Res.

[61] White B, Saunders F, Scadden L, Bach-Y-Rita P, Collins C (1970). Seeing with the skin. Percept Psychophys.

[62] Wiesenfeld K, Moss F (1995). Stochastic resonance and the benefits of noise: from ice ages to crayfish and SQUIDs. Nature.

[63] Witteveen H, Rietman H, and Veltink P. Vibrotactile grasping force and hand aperture feedback for myoelectric forearm prosthesis users. Prosthet Orthot Int. 2015;39(3):204-12. 10.1177/0309364614522260 24567348

[64] Zackowski KM, Dromerick AW, Sahrmann SA, Thach WT, Bastian AJ (2004). How do strength, sensation, spasticity and joint individuation relate to the reaching deficits of people with chronic hemiparesis?. Brain.

