# Erratum to: Supplemental vibrotactile feedback control of stabilization and reaching actions of the arm using limb state and position error encodings

**DOI:** 10.1186/s12984-017-0281-7

**Published:** 2017-07-10

**Authors:** Alexis R. Krueger, Psiche Giannoni, Valay Shah, Maura Casadio, Robert A. Scheidt

**Affiliations:** 1 Biomedical Engineering, Marquette University, Milwaukee, WI USA; 2 Informatics, Bioengineering, Robotics and Systems Engineering, University of Genova, Genoa, Italy; 3 Robotics, Brain and Cognitive Science, Italian Institute of Technology, Genoa, Italy; 4 Physical Medicine and Rehabilitation, Northwestern University Feinberg School of Medicine, Chicago, IL USA; 5 Sensory Motor Performance Program, Rehabilitation Institute of Chicago, Chicago, IL USA; 6 Neurology, Medical College of Wisconsin, Wauwatosa, WI USA; 7 Neuromotor Control Laboratory, Department of Biomedical Engineering, Marquette University, Olin Engineering Center, 206, P.O. Box 1881, Milwaukee, WI 53201-1881 USA

## Erratum

The original article [1] contained omissions mistakenly carried forward by the Production department handling this journal; these omissions related to missing ‘λ’ symbols throughout the article body, thus inaccurately representing the meaning of the associated text.

The article has now been updated to include these symbols in the appropriate sections of text to accurately reflect the conveyed implications.
